# Single-cell transcriptomics reveals maturation of transplanted stem cell–derived retinal pigment epithelial cells toward native state

**DOI:** 10.1073/pnas.2214842120

**Published:** 2023-06-20

**Authors:** Bhav Harshad Parikh, Paul Blakeley, Kakkad Regha, Zengping Liu, Binxia Yang, Mayuri Bhargava, Daniel Soo Lin Wong, Queenie Shu Woon Tan, Claudine See Wei Wong, Hao Fei Wang, Abdurrahmaan Al-Mubaarak, Chai Chou, Chui Ming Gemmy Cheung, Kah Leong Lim, Veluchamy Amutha Barathi, Walter Hunziker, Gopal Lingam, Tim Xiaoming Hu, Xinyi Su

**Affiliations:** ^a^Institute of Molecular and Cell Biology, Agency for Science, Technology and Research, Singapore 138673, Singapore; ^b^Department of Ophthalmology, Yong Loo Lin School of Medicine, National University of Singapore, Singapore 119228, Singapore; ^c^Singapore Eye Research Institute, Singapore 169856, Singapore; ^d^Department of Ophthalmology, National University Hospital, Singapore 119074, Singapore; ^e^Lee Kong Chian School of Medicine, Nanyang Technological University, Singapore 308232, Singapore; ^f^Academic Clinical Program in Ophthalmology, Duke-NUS Medical School, Singapore 169857, Singapore; ^g^Department of Physiology, Yong Loo Lin School of Medicine, National University of Singapore, Singapore 117593, Singapore; ^h^Institute of Health Innovation and Technology, National University of Singapore, Singapore 119276, Singapore

**Keywords:** pluripotent stem cell, retinal pigment epithelium, cell transplantation, transcriptome

## Abstract

Transplantation of stem cell–derived retinal pigment epithelial (RPE) cells is considered a viable therapeutic option for age-related macular degeneration (AMD). Several landmark Phase I/II clinical trials have demonstrated safety and tolerability of RPE transplants in AMD patients, albeit with limited efficacy. Currently, there is limited understanding of how the recipient retina regulates the survival, maturation, and fate specification of transplanted RPE cells. To address this, we transplanted stem cell–derived RPE into the subretinal space of immunocompetent rabbits for 1 mo and conducted single-cell RNA sequencing analyses on the explanted RPE monolayers, compared to their age-matched in vitro counterparts. We observed an unequivocal retention of RPE identity, and a trajectory-inferred survival of all in vitro RPE populations after transplantation. Furthermore, there was a unidirectional maturation toward the native adult human RPE state in all transplanted RPE, regardless of stem cell resource. Gene regulatory network analysis suggests that tripartite transcription factors (*FOS*, *JUND*, and *MAFF*) may be specifically activated in posttransplanted RPE cells, to regulate canonical RPE signature gene expression crucial for supporting host photoreceptor function, and to regulate prosurvival genes required for transplanted RPE’s adaptation to the host subretinal microenvironment. These findings shed insights into the transcriptional landscape of RPE cells after subretinal transplantation, with important implications for cell-based therapy for AMD.

## Significance

Preclinical evaluation of retinal pigment epithelial (RPE) cell–based therapy has largely been dependent on qualitative in vivo ophthalmic imaging techniques and ex vivo histological characterization of transplanted cells. However, these readouts do not provide a comprehensive picture of gene expression changes at single-cell resolution. In this study, we transplanted RPE monolayers in the subretinal space of immunocompetent rabbits and performed single-cell RNA sequencing. We observed unequivocal maturation toward the native adult human RPE state, and identified key regulons activated upon transplantation. These key regulons may confer RPE canonical functions important for retinal homeostasis and promote cell survival in vivo. These findings shed insights into the behavior of RPE after subretinal transplantation, with important implications for cell-based therapy for age-related macular degeneration.

Age-related macular degeneration (AMD) is the leading cause of irreversible blindness and visual impairment in the elderly population, affecting 196 million people in 2020, with a potential of reaching 288 million in 2040 (1). Atrophic AMD is characterized by progressive and significant retinal pigment epithelial (RPE) cell loss that leads to photoreceptor degeneration. Currently, there is no effective treatment for atrophic AMD with RPE cell loss (2, 3). With the advent of embryonic stem cell (ESC) and induced pluripotent stem cell (iPSC) technology, cell-based RPE therapy is a promising approach for AMD treatment (4). By transplanting healthy stem cell–derived RPE cells to replace diseased host cells, it is envisaged that host retinal function can be reestablished to provide durable functional recovery for patients (5).

RPE is a single layer of pigmented and polarized epithelium, required to support retinal photoreceptor function. They phagocytose the shed lipid-rich photoreceptor outer segments (POS) (necessary for photo-transduction), metabolize, and recycle components back to photoreceptors for efficient maintenance of the visual cycle. The RPE also plays key roles in forming the outer blood–retina barrier, regulating fluid transport, cytokine release, and ionic balance, all of which are functions critical for retinal homeostasis (6). Recent landmark Phase I/II trials studies have demonstrated the safety of different sources of human pluripotent stem cell (hPSC)–derived RPE transplantation in AMD patients (7–11) However, key challenges remain to be addressed before RPE replacement therapy can be adopted as the standard-of-care in the treatment of AMD. This includes establishing robust methods to evaluate transplanted RPE survival, functional integration with overlying host retinal tissue in situ, and interaction with host-immune system, while avoiding the risk of dedifferentiation or pluripotency, to ultimately support vision recovery.

Current preclinical studies rely largely on conventional in vivo ophthalmic imaging modalities such as fundus photography (FP), fundus autofluorescence (FAF), optical coherence tomography (OCT), and electroretinography (ERG) for outcome measurements related to the morphology or function of the eye, and ex vivo histological characterization of surviving transplanted cells to provide qualitative evaluations on RPE transplantation outcomes (12). However, these readouts are unable to interrogate gene expression, cellular signaling, and function, in an unbiased fashion and at the single-cell resolution. Recent single-cell RNA sequencing (scRNA-seq) of in vitro hPSC–derived RPE have identified the presence of multiple subpopulations (13–15). Therefore, a single-cell approach is important to allow us to understand and monitor the survival, maturation, and fate specification of transplanted RPE cells, with the ultimate goal of understanding key transplantation parameters that might prime clinical outcomes for success.

In this study, we generated mature and functional RPE cells from two hPSC sources: ESC and skin-derived iPSC (abbreviated as: ESC-RPE and iPSC-RPE, respectively). The RPE were subretinally transplanted in a monolayer format into immunocompetent rabbit eyes and followed up in vivo for 30 d. We sought to understand changes in transcriptome at the single-cell level in these two RPE lines after transplantation. After transplantation, we observed maintenance of specific RPE gene expressions involved in photoreceptor homeostasis (visual cycle, phagocytosis, growth factors), and further activation of genes involved in oxidative and lipid metabolism, and ECM organization. We observed a unidirectional maturation trajectory toward the native adult human RPE state in all transplanted RPE cells, regardless of stem cell resource. Reconstruction of the gene regulatory network (GRN) of the stem cell–derived RPE posttransplantation enabled us to identify activation of key regulons that are likely to enable RPE survival, maturation, and integration into an immunocompetent subretinal space.

## Results

### Generation of Mature and Functional Stem Cell-Derived RPE Monolayers.

**Fig. 1.**
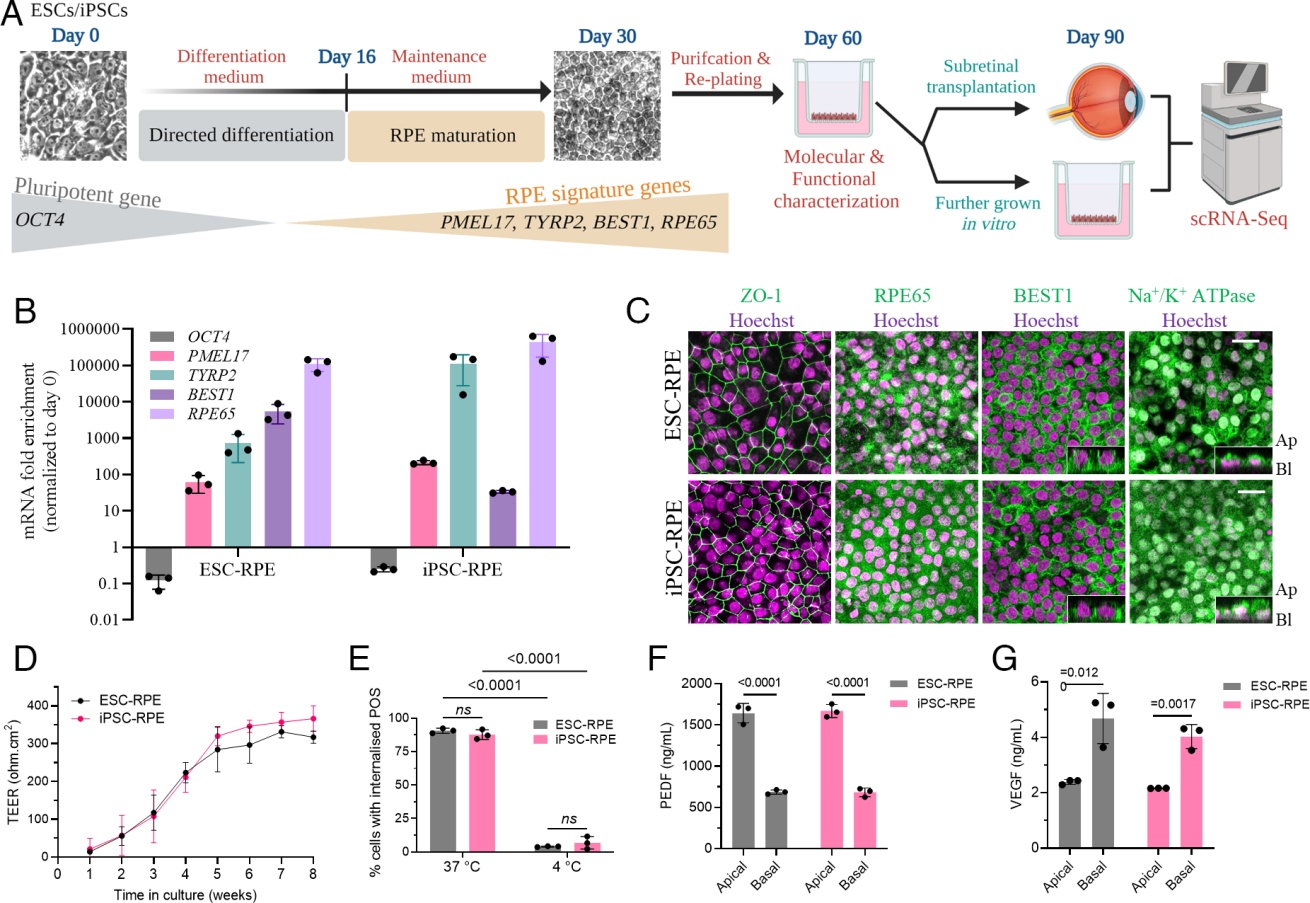
Generation and characterization of ESC and iPSC differentiated RPE. (*A*) Schematic of the study workflow. RPE cells were differentiated and replated on Transwell inserts on day 30. RPE monolayer was transplanted in rabbit eyes at day 60 and extracted for scRNA-seq at day 90. RPE maturity in culture was characterized at day 60 on Transwell inserts. (*B*) mRNA expression of pluripotent gene*, OCT4*, was down-regulated after differentiation, while RPE signature genes (*PMEL17*, *TYRP2*, *BEST1*, and *RPE65*) were up-regulated in both day 60 ESC- and iPSC-RPE. (*C*) Immunofluorescence (IF) of RPE-specific proteins, ZO-1, RPE65, BEST1, and Na^+^/K^+^ ATPase. *Inset* in BEST1 and Na^+^/K^+^ ATPase panels show the basolateral and apical localizations, respectively. Representative images of both ESC- and iPSC-RPE from three independent experiments are shown. (Scale bar, 20 µm.) (*D*) Transepithelial electrical resistance (TEER) of each RPE line from day 30 onward was monitored over 8 wk and showed a similar progressive increase in resistance. (*E*) Flow cytometry was used for the measurement of percentage (%) cells that phagocytosed FITC-labeled photoreceptor outer segments (POS) at 37 °C and 4 °C. Phagocytosis was inhibited at 4 °C. (*F* and *G*) Polarized secretion of PEDF and VEGF from RPE lines was studied using ELISA and demonstrated predominantly apical and basal secretion, respectively. For *B* and *D*–*G*, data represent mean ± SD of three replicates. Statistical analysis for *E* was performed using one-way ANOVA followed by Tukey’s honest significance difference (HSD) post hoc test, and for *F* and *G* using two-tailed Student’s unpaired *t* test. *ns* = not significant. The schematic in *A* was illustrated using BioRender (https://biorender.com).

RPE cells were generated from two hPSC sources (ESC and skin fibroblast–derived iPSC) with a published differentiation protocol (16, 17) (Fig. 1*A*). After the 16-d differentiation, ESC- and iPSC-derived RPE were allowed to mature until day 30, before purifying and replating on a polyester Transwell inserts for another 30 d to promote maturation and apicobasal polarization. We confirmed the loss of the pluripotency marker (*OCT4*), and the gain of the RPE-specific markers (*PMEL17, TYRP2*, *BEST1* and *RPE65*) (18) in day 60 ESC- and iPSC-RPE, compared to its day 0 hPSC (Fig. 1*B*). Consistent with in vitro maturation, both day 60 RPE lines expressed RPE-specific proteins with appropriate subcellular localization of zonula occludens-1 (ZO-1), retinoid isomerohydrolase (RPE65), bestrophin-1 (BEST1), and sodium-potassium ATPase pump (Na^+^/K^+^ ATPase) (Fig. 1*C*).

We proceeded to confirm that both RPE lines were able to perform RPE-specific functions such as maintenance of outer blood–retinal barrier by measuring transepithelial electrical resistance (TEER), support of photoreceptor function via phagocytosis of shed POS, and polarized secretion of cytokines such as pigment epithelium–derived factor (PEDF) and vascular endothelial growth factor (VEGF) – all of which are crucial for the maintenance of retinal homeostasis (6). TEER of replated day 30 ESC- or iPSC-RPE monolayer progressively increased during the first 6 wk of culture (day 30 to 72) on a Transwell insert and maintained between week 6 and 8 (ESC-RPE: 317 ± 16 ohms·cm^2^, *n *= 3; iPSC-RPE: 366 ± 33 ohms·cm^2^, *n *= 3) (Fig. 1*D*). Both ESC- and iPSC-RPE lines at day 60 demonstrated >87% uptake of fluorescein-isothiocyanate (FITC)-POS at 37 °C (ESC-RPE: 90.6 ± 1.9%, *n *= 3; iPSC-RPE: 87.7 ± 3.6%, *n *= 3) (Fig. 1*E*). Day 60 RPE cells also demonstrated polarized secretion of cytokines PEDF and VEGF, with predominant presence in the apical and basal chambers of the Transwell insert, respectively (Fig. 1 *F* and *G*). This confirms that in vitro matured ESC- and iPSC-RPE demonstrate functional properties similar to those expected of a healthy adult human RPE (19).

### Subretinal Transplantation of RPE Monolayers in Immunocompetent Rabbits.

**Fig. 2.**
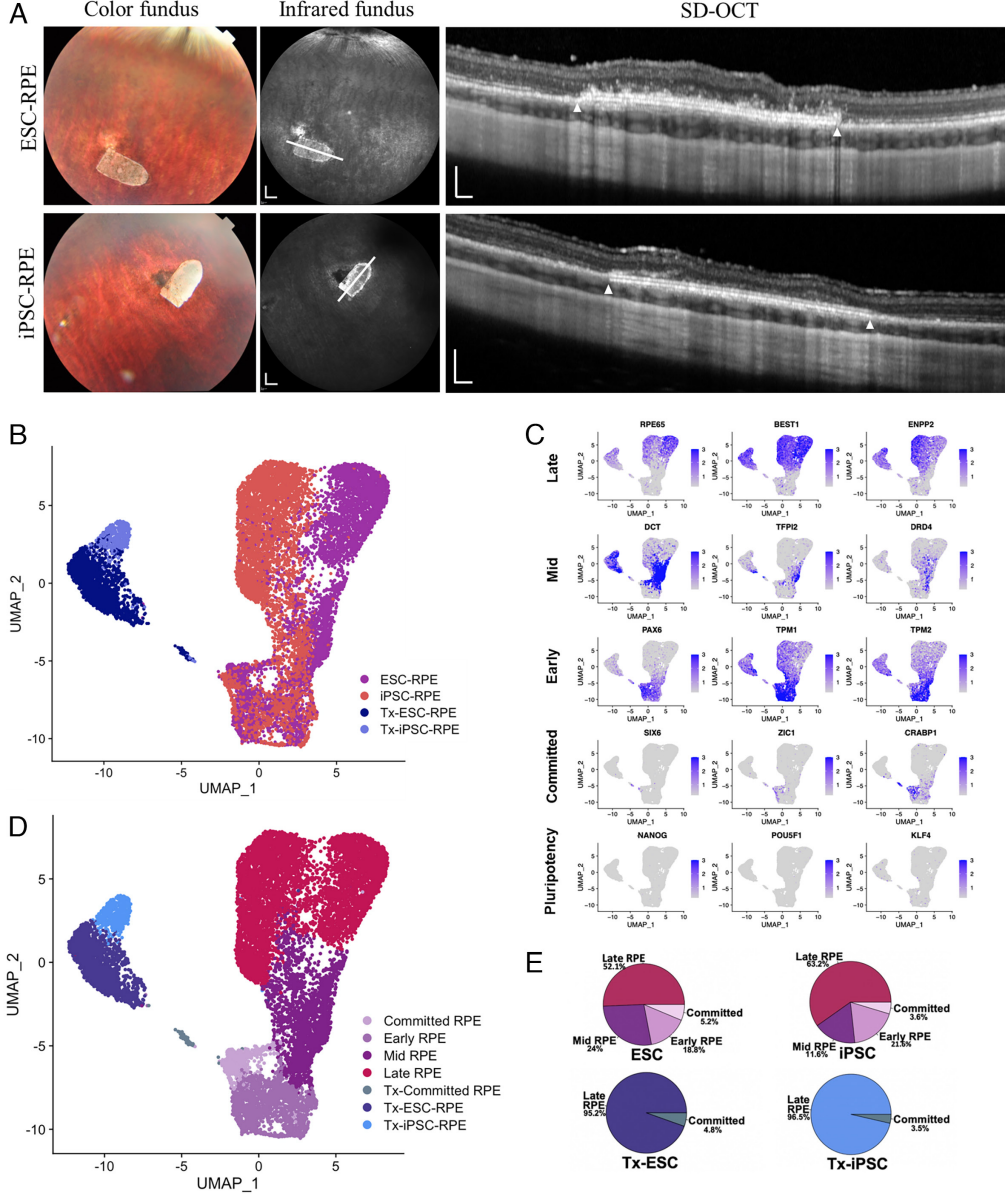
Transcriptomic profiling of ESC- and iPSC-RPEs before and after subretinal transplantation. (*A*) In vivo ophthalmic imaging of subretinally transplanted day 60 RPE monolayer implants (Tx-ESC-RPE: *n *= 3; Tx-iPSC-RPE: *n *= 3) into immunocompetent Dutch belted rabbits at 30 d postsurgery. The imaging modalities of color fundus, infrared fundus and spectral domain-optical coherence tomography (SD-OCT) were used. The bullet-shaped RPE implants were visible in the color fundus images. The white lines indicated positions at which SD-OCT cross-section images were taken. The white triangles indicated the boundaries of the implants on SD-OCT. (Scale bar, 2 mm in infrared fundus, 200 µm in SD-OCT images.) (*B*) UMAP dot plot visualization of single-cell transcriptomes of all 13232 RPE cells, colored by sample library. In vitro (before transplantation): ESC-RPE and iPSC-RPE (*n *= 1 each). Posttransplantation: Tx-ESC-RPE and Tx-iPSC-RPE (*n *= 3 each). (*C*) Normalized expression of genes corresponding to different RPE maturity states (‘Pluripotent’, ‘Committed’, ‘Early’, ‘Mid’ and ‘Late’) are plotted onto the UMAP in *B*. (*D*) UMAP plot showing systemic annotation of RPE maturity states, colored by unsupervised clustering based on UMAP in *B*. (*E*) Proportion of cells within each subpopulation before and after transplantation for ESC- and iPSC-RPE lines.

Next, we investigated ESC- and iPSC-RPE’s ability to survive posttransplantation in a large-eyed animal model. RPE monolayers were allowed to mature in vitro on Transwell inserts until day 60 (Fig. 1*A*), before transplantation (ESC-RPE, *n *= 3; iPSC-RPE, *n *= 3) into the subretinal space of immunocompetent Dutch belted rabbit eyes as published previously (20, 21). In all six rabbit eyes, the 1 × 2 mm RPE monolayer grafts remained in position, with well-healed retinotomy sites and reattached retina, when examined at 30 d after transplantation. (Fig. 2*A* and *SI Appendix*, Fig. S1). The retinal structure overlying the RPE monolayer grafts (*n *= 6) was preserved on spectral domain-optical coherence tomography (SD-OCT) images. Occasional presence of hyperreflective foci above the RPE grafts were observed for both ESC- and iPSC-RPE, possibly indicating a mild and localized inflammatory reaction (22, 23) from the host rabbit. However, the absence of persistent subretinal fluid, retinal atrophy, or vitreous inflammation suggests the absence of a severe inflammatory response (24). Consistent with this, we observed preservation of global retinal function, as demonstrated by the maintenance of both a- and b-wave amplitudes and waveforms in both photopic and scotopic conditions on full field ERG at 30 d postsurgery, comparable to the contralateral nonoperated eye (*SI Appendix*, Fig. S2).

### ScRNA-seq Analysis Comparing RPE Cells Pretransplantation and Posttransplantation.

We next investigated single-cell gene expression changes in transcriptomes of subretinally transplanted RPE (Tx-RPE), compared to their age-matched day 90 in vitro counterparts. Day 90 Tx-RPE monolayers (Tx-ESC-RPE: *n *= 3; Tx-iPSC-RPE: *n *= 3) were isolated from enucleated rabbit eyes’ retina, and dissociated as per published protocol for downstream scRNA-seq (25). A total of 13,232 individual RPE cells (in vitro RPE: 10,772 cells; Tx-RPE: 2,460 cells) with high quality controlled scRNA-seq data were profiled (*SI Appendix*, Figs. S3–S6 and Table S1). Due to our stringent QC thresholds, all cells from a single Tx-iPSC-RPE sample (#3) were excluded (*SI Appendix*, Table S2). The 13,232 cells were projected into two dimensions by Uniform Manifold Approximation and Projection (UMAP) (26) using their global gene expression profiles (Fig. 2*B*). Unexpectedly, we observed a major separation between the day 90 in vitro and Tx-RPE cells. This suggested that the host retinal microenvironment exerted a strong influence on the transplanted RPE cells. Nevertheless, all 2,460 Tx-RPE cells, consisting of both ESC- and iPSC-RPE, grouped tightly together despite being transplanted into five different rabbit eyes. This consistent shift in global transcriptome posttransplantation (*SI Appendix*, Fig. S3*D*) strongly suggests that subretinal transplantation of RPE induces a similar shift in its transcriptional landscape regardless of its hPSC source.

Stem cell–derived RPE lines generated using various differentiation protocols are known to be heterogeneous, consisting of RPE cells at various stages of maturation (13–15). To ascertain whether our ESC- and iPSC-RPE lines also presented such heterogeneity, we looked at the expression profiles of several genes known to be differentially expressed through RPE maturation (Fig. 2*C*). We then performed a priori clustering of the full transcriptome of all cells, as shown on the UMAP (Fig. 2*D*), and observed that both in vitro and Tx-RPE were organized in a gradient of “matureness” from the least mature (expressing *SIX6*), to the most mature (expressing *RPE65*). Within the in vitro RPE clusters on the UMAP, there was a clear progression from least mature (termed ‘committed’) RPE (expressing *SIX6*, *ZIC1*, and *CRABP1*), toward a more mature RPE—termed ‘early’, ‘mid,’ to ‘late’ RPE (expressing *RPE65*, *BEST1*, and *ENPP2*). In addition, we observed a divergence between the ‘late’ RPE clusters of in vitro iPSC-RPE and ESC-RPE indicating some line-specific differences. The relative abundance of RPE cells at different maturity states was also quantified. The majority of in vitro ESC- and iPSC-RPE consisted of ‘late’ RPE with 52.1% and 63.2% respectively, with varying percentages of less mature ‘committed’, ‘early’ and ‘mid’ RPE populations (Fig. 2*E*). We observed that this heterogeneity of RPE states reduced significantly after transplantation. In both Tx-ESC-RPE and Tx-iPSC-RPE, majority of the cells belonged to the ‘late’ RPE subpopulation, comprising of 95.2% and 96.5%, respectively. This suggests the unidirectional maturation of transplanted RPE within the subretinal space, presumably to facilitate acquisition of additional RPE functional properties to support retinal homeostasis. Importantly, we confirmed that both our in vitro and Tx-RPE cells did not express pluripotent genes, such as *NANOG*, *POU5F1,* and *KLF4* (Fig. 2*C*). This is crucial, as the presence of undifferentiated pluripotent cells carries the risk of tumorigenicity, leading to cell therapy failure (27).

### Transplanted Stem Cell–Derived RPE Evolve toward the Adult Human RPE.

**Fig. 3.**
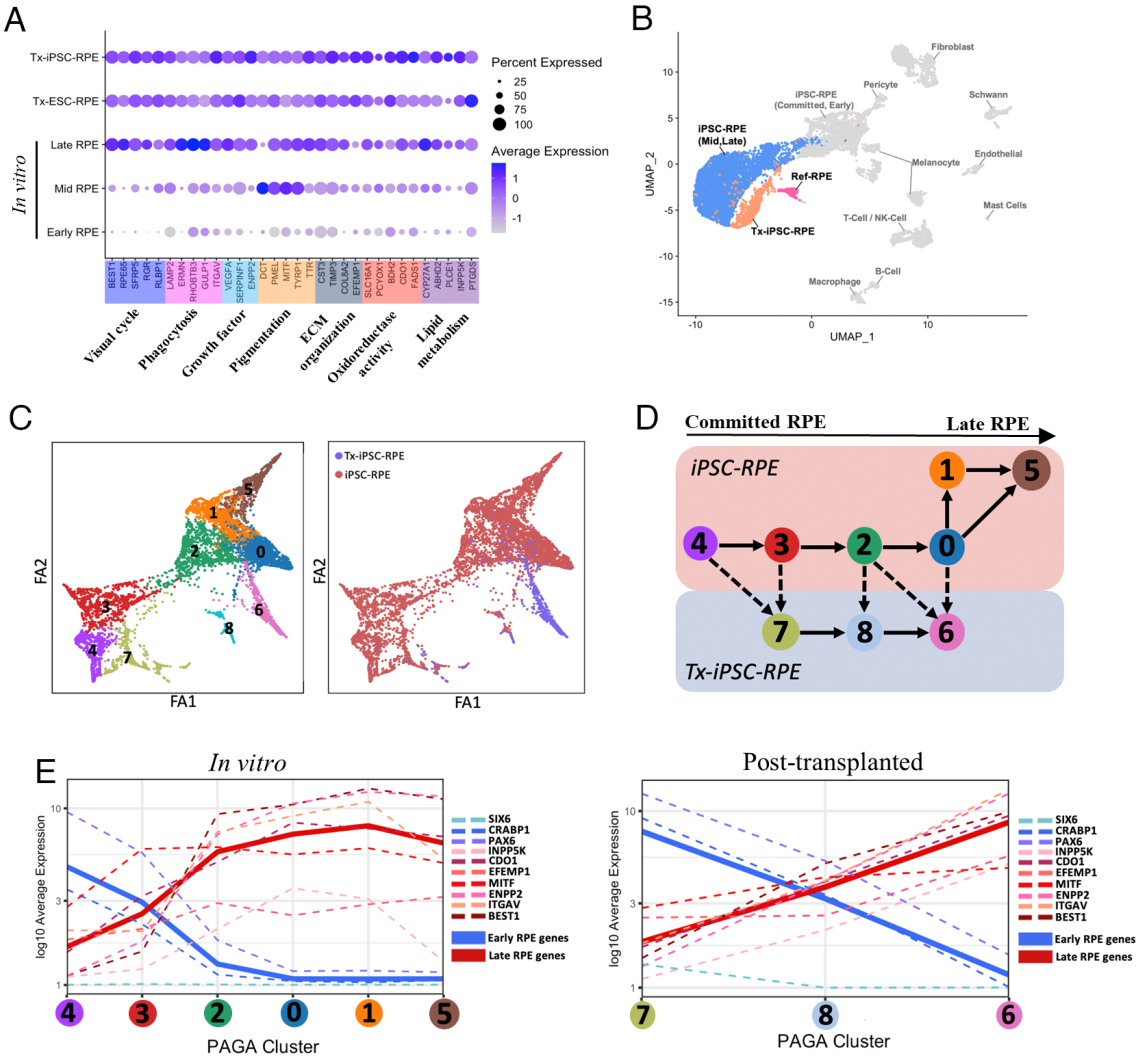
Differential maturation trajectory of RPE before and after transplantation. (*A*) Bubble plot representation of single-cell expression profiles from different RPE subpopulations for selected genes, representative of seven RPE-specific functional categories. Frequencies of cells expressing the respective gene (percent expressed) are shown with size of dots. Levels of gene expression per cell are shown with color gradients. (*B*) UMAP dot plot showing the similarity of iPSC-RPE and Tx-iPSC-RPE relative to various cell types present in healthy adult human RPE-choroid tissue reference scRNA-seq dataset. (*C*) Force-directed graph showing the trajectory of iPSC-RPE and Tx-iPSC-RPE subpopulations before and after transplantation. (*C*, *Left*) Dots are colored by results of a prior clustering of iPSC-RPE and Tx-iPSC-RPE. (*C*, *Right*) Dots are colored by before or after transplantation. (*D*) Schematic showing the result from PAGA analyses, each circle indicates one cluster in the *Left* panel of *C*. Each arrow joining two clusters indicates that they are highly similar in their global transcriptomic profile and are adjacent in the maturation trajectory. Solid arrows represent maturation trajectory within the same condition, and the dashed arrows represent the inferred maturation trajectory starting from in vitro cluster toward their nearest posttransplanted cluster. (*E*) Using the maturation trajectory information obtained in analyses in *D*, the average expression of selected RPE-specific genes within a cluster was plotted as a dashed curve onto the maturation trajectory. The thick solid curve is the average of ‘early’ RPE markers (blue) and ‘late’ RPE markers (red). These analyses were repeated for both in vitro and posttransplanted iPSC-RPE.

Native RPE has various functions in vivo, for which transplanted RPE cells need to acquire in situ to maintain retinal homeostasis. To interrogate if both the in vitro matured RPE and Tx-RPE do indeed acquire these known RPE functional properties, we grouped well-studied RPE genes into seven canonical RPE function categories (28) and investigated their expression levels across the different maturity states (Fig. 3*A*). We observed that in vitro RPE had a gradual acquisition of gene expression in all categories of RPE function, as it transited from ‘early’ to ‘late’ RPE. Interestingly, genes involved in ‘melanin synthesis’ (*DCT*, *PMEL*, *MITF,* and *TYRP2*) peaked in ‘mid’ RPE. This is consistent with reports that melanin synthesis peaks early in embryogenesis, and is thereafter switched off (29). Importantly, genes such as *RPE65* and *RLBP1* (involved in the visual cycle), and *ERMN* and *RHOBTB3* (involved in phagocytosis) had maximal expression in ‘late’ in vitro RPE. In contrast, Tx-RPE cells had a unique trend toward increased expression of genes involved in the following pathways of ‘ECM organization’ (*TIMP3* and *EFEMP1*), ‘oxidoreductase activity’ (*BDH2* and *CD01*), and ‘lipid metabolism’ (*ABHD2* and *PTGDS*). Increased expression of genes in these pathways suggests acquisition of RPE secretory functions important for the formation of interphotoreceptor matrix (30), protection from oxidative stress in-vivo after exposure to visible light and high oxygen tension (15), and processing of lipid-rich host POS during phagocytosis (31), respectively. In summary, both Tx-ESC- and Tx-iPSC-RPE demonstrated attributes of canonical RPE gene expression that were increased upon transplantation suggesting in vivo functional integration with host retinal tissue.

To substantiate this positive influence of the retinal microenvironment on the Tx-RPE monolayers, we compared their transcriptomic profile to a reference healthy adult human RPE-choroid scRNA-seq dataset (32). UMAP of the batch integrated data showed that both Tx-iPSC- and Tx-ESC-RPE, unlike in vitro day 90 RPE, were most similar to reference adult human RPE (labeled ‘Ref_RPE’) (Fig. 3*B* and *SI Appendix*, Fig. S7*A*). This confirmed the favorable maturation of Tx-RPE toward its native RPE counterpart found in healthy human donor eyes.

### Single-Cell Trajectories of RPE Cell Subpopulations after Transplantation.

Similar to published data (13–15), we also observed various subpopulations in our in vitro cultured stem cell–derived RPE. However, it remains unknown, how these subpopulations of RPE cells evolve transcriptionally after subretinal transplantation. To interrogate this, we performed pseudotime developmental trajectory analysis on both in vitro and Tx-RPE cells using partition-based graph abstraction (PAGA) (33) to analyze the connectivity between the clusters, and infer trajectories of in vitro subpopulations transitioning to Tx-RPE cells upon transplantation into the rabbit subretinal space (Fig. 3 *C* and *D* and *SI Appendix*, Fig. S7 *B* and *C*). To ascertain the direction of maturation within this force-directed graph, we used representative genes from the seven functional categories previously used (Fig. 3*A*), to plot gene expression values as curves over the pseudotime trajectory. For in vitro iPSC-RPE, these RPE functional genes reached peak expression levels in cluster ‘0’, which coincidentally served as a central point before diverging into in vitro clusters ‘1’ and ‘5’, and posttransplanted cluster ‘6’ (Fig. 3*E*). This trend was observed in ESC-RPE, whereby there was a central cluster ‘3’ that served as a common point for the in vitro and posttransplanted RPE before diverging into in-vitro cluster ‘4’ and ‘2’, and posttransplanted cluster ‘1’ (*SI Appendix*, Fig. S7*D*).

We further dissected these divergent clusters, in an effort to unravel the differential effects of maturing cultured RPE cells in vitro versus transplanted RPE in vivo. Pathway enrichment analysis was performed, comparing the ‘terminal’ and hence presumed most mature in vitro and posttransplanted clusters for both ESC- and iPSC-RPE (i.e., cluster ‘1’ vs. ‘2’ for ESC-RPE; cluster ‘6’ vs. ‘5’ for iPSC-RPE) (*SI Appendix*, Fig. S8). Enriched pathways were broadly categorized into those involved in ‘RPE function’, ‘signaling’, ‘metabolism’, ‘immune response’ and ‘gene regulation’. For both Tx-ESC- and Tx-iPSC-RPE, although majority of the up-regulated pathways were found to be involved in canonical RPE functions (‘collagen secretion’, ‘response to light stimulus’ and ‘cell substrate junction’), there were some line-specific differences observed in response to transplantation. Particularly for Tx-iPSC-RPE, we observed increased pathways involved in RPE maturation (‘regulation of hormone levels’, ‘pigment granule’ and ‘retinoic acid metabolic process’) that were not observed for Tx-ESC-RPE. This suggests that subretinal transplantation has a general positive influence on RPE maturation, with minor cell line-specific differences that may influence the ultimate transplantation outcome.

### GRN Analysis Reveals the Effect of Transplantation.

**Fig. 4.**
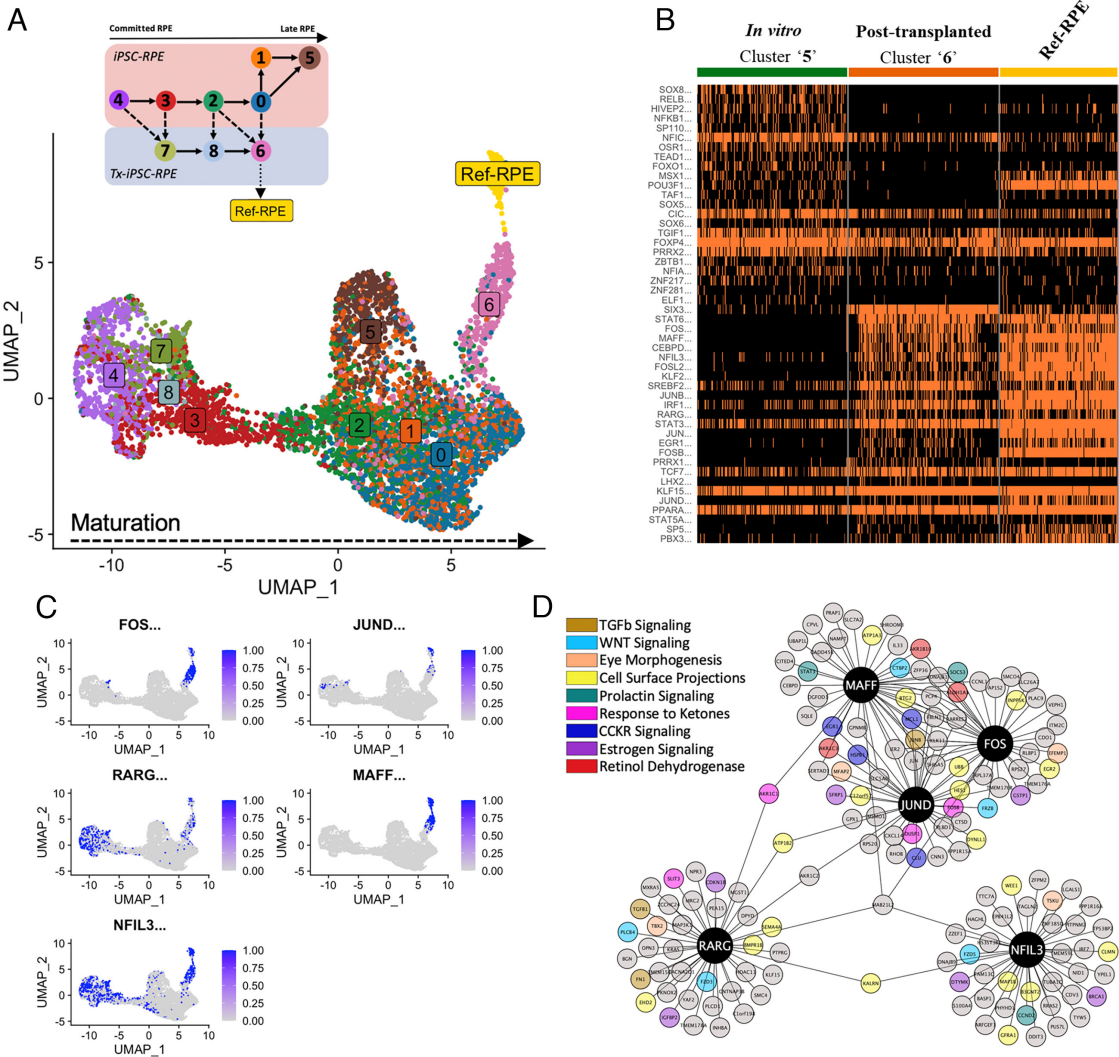
Transcription factor regulon analyses of iPSC-RPE. (*A*) UMAP dot plot based on binarized AUC scores showing transcription factor regulon status of the in vitro and posttransplanted iPSC-RPE, in comparison to a reference dataset of healthy adult human RPE, termed as Ref-RPE. Cluster ‘6’ of Tx-iPSC-RPE is closest to Ref-RPE. The dots are colored by PAGA trajectory states (*Inset* schematic). (*B*) Heatmap showing regulons with elevated activity (Likelihood Ratio Test: *P*_adj_ < 0.05) between ‘late’ Tx-iPSC-RPE (cluster ‘6’) and ‘late’ iPSC-RPE (cluster ‘5’), based on binarized AUC scores. (*C*) Binarized AUC scores of five highly active regulons shared between Tx-iPSC-RPE and Ref-RPE across all cells. (*D*) Top 40 target genes with the highest GRNBoost scores for the five regulons concurrently active in Tx-iPSC-RPE and Ref-RPE. The genes are further categorized into pathway functions modulated by the five identified regulons.

The transcriptional state of a cell is regulated by a GRN, consisting of a group of transcription factors and their target genes. It has not been previously reported how GRN changes after subretinal transplantation of RPE. Therefore, we performed single-cell regulatory network interference and clustering (SCENIC) (34) analysis to determine the GRN associated with the transition of transplanted RPE toward adult human RPE (Ref_RPE) (Fig. 3*B*), and away from the in vitro cluster. The GRN obtained revealed a gradient of maturation from left to right for both Tx-iPSC- and Tx-ESC-RPE (Fig. 4 *A* and *B* and *SI Appendix*, Fig. S9). In comparison to their in vitro counterparts, Tx-iPSC-RPE (Cluster ‘6’) displayed much closer clustering to healthy adult human RPE reference (i.e., ‘Ref_RPE’), indicating high similarity in those cell populations compared to their divergent in vitro counterparts (iPSC-RPE: Cluster ‘5’). Similarly, the Tx-ESC-RPE (Cluster ‘1’) located in close proximity to Ref_RPE. Upon comparing iPSC-RPE with ESC-RPE (before and after transplantation), we observed the highest number of overlapping transcription factors in Tx-iPSC-RPE with Ref_RPE (*SI Appendix*, Fig. S10*A*). Therefore, we focused on regulons observed in Tx-iPSC-RPE and Ref_RPE (*SI Appendix*, Fig. S10*B*). We detected various active, canonical RPE transcription factors (such as *KLF2*, *FOSL2*, *PRRX1*) (35, 36) to be active only upon transplantation in Tx-iPSC-RPE but not in in vitro iPSC-RPE suggesting that Tx-iPSC-RPE may better recapitulate adult human RPE, at the GRN level.

To understand the GRNs responsible for the similarity of Tx-RPE to Ref_RPE, we focused on the five overlapping highly active transcription factors (*FOS*, *JUND*, *RARG*, *MAFF* and *NFIL3*) present in Tx-ESC-RPE, Tx-iPSC-RPE and Ref_RPE. These transcription factors, particularly *FOS*, *JUND* and *MAFF*, were only enriched in the Tx-iPSC-RPE’s most ‘advanced’ posttransplanted cluster (‘6) and Ref_RPE (Fig. 4*C*), suggesting their unique and important roles for RPE functions in vivo. To validate the GRNs, we selected *FOS* as a representative transcription factor and analyzed its protein expression and localization in transplanted RPE cells that were detected through costaining of human-specific antigen (TRA-1-85) and RPE-specific proteins (OTX2 and RPE65). Indeed, we observed nuclear localization of c-FOS (protein expressed by *FOS* gene) in transplanted RPE cells suggesting its activation in vivo in rabbit eyes (*SI Appendix*, Fig. S11).

We subsequently identified top 40 downstream target genes of FOS, *JUND,* and *MAFF*. These genes were related to nine different pathways including ‘cell surface projection’, ‘eye morphogenesis’ and ‘retinol dehydrogenase’ that could contribute to RPE function in vivo and allow maintenance of RPE cell identity and integration with host photoreceptors (Fig. 4*D*). Other pathways identified include ‘prolactin signaling’, ‘CCKR signaling’ and ‘estrogen signaling’ which are likely to be involved RPE cell survival (37–39). Taken together, the identified GRNs reveal unique factors that may potentially be involved in RPE cell survival in vivo, as well as in functions crucial for maintaining photoreceptor homeostasis to support host retinal tissue.

## Discussion

RPE cell replacement has emerged as a promising therapy for AMD patients. Many studies have demonstrated reliably that hPSC-derived RPE cultures in vitro, closely resembling healthy human RPE (40), can be delivered safely into the subretinal space (5). Current preclinical studies evaluating outcomes of RPE transplants, have largely relied on qualitative evaluations using traditional in vivo ophthalmic imaging techniques such as FP, OCT, and FAF. Evidence of cell survival and integration is further corroborated by ex vivo histology as an outcome measure (41, 42). However, these conventional methods of investigations are low throughput, and do not provide in-depth insights into how RPE heterogeneity, recently reported to be present in in vitro stem cell–derived RPE cultures (13–15) may affect RPE transplant outcomes. More importantly, these readouts also do not provide a comprehensive picture of single-cell gene expression changes in transplanted RPE cells. Last, these traditional methods are dependent on probing known RPE-related functions and does not allow a priori identification of transcriptomic changes in response to subretinal transplantation. Neither do they enable us to understand how RPE cells adjust, adapt, and evolve upon subretinal transplantation in vivo, at a single-cell resolution.

To address these questions, we investigated how this known heterogeneity of in vitro ESC- and iPSC-RPE impacts RPE transplant outcomes using scRNA-seq. We demonstrated the consistent transition and maturation of a heterogenous in vitro RPE cultures (consisting of 50 to 65% ‘late’ RPE), toward a mostly homogenous ‘late’ RPE population (>95%) in the subretinal microenvironment of rabbit eyes after transplantation. This held true regardless of the type of hPSC resource used, and importantly, was validated in five separate rabbit eyes. In this study, we chose to transplant day 60 RPE monolayers and let them further mature in the subretinal microenvironment for another 30 d, before comparing their gene expression profiles with age-matched day 90 in vitro RPE cultures, accepted as “matured RPE cultures” (43). Transplantation of RPE monolayer has been shown to improve survival, promote, and support RPE maturation in animal models, because the RPE cells would have already achieved polarization preimplantation (42, 44). This enables RPE monolayers to readily adapt to the in vivo environment posttransplant, and perform RPE functions more readily, without requiring additional time for repolarization, unlike in RPE cell suspension transplants. Efficient RPE cell recovery from the explanted RPE scaffolds in our study was enabled using our published RPE monolayer transplant surgical technique (20, 45). Thus, we were able to retrieve and sequence a total number of 2,460 transplanted RPE cells, compared to a recent study, whereby only 65 RPE cells were retrieved from subretinal RPE cell suspension transplant in two rabbits (14). However, one limitation of our approach is the inability to accurately quantify percentage of RPE cell survival on the scaffold in vivo, due to cell loss during downstream processing steps such as scaffold retrieval and preparation of single-cell suspension. Nonetheless, using this technique, we demonstrated that transplantation of stem cell–derived RPE monolayer is able to survive and mature toward native adult human RPE phenotype.

RPE transplant outcomes have been focused on evaluating the ability of RPE to integrate with host retinal tissue. This includes supporting the functions of overlying photoreceptors using standard ophthalmic imaging modalities (12). In this study, we utilized a unique scRNA-seq approach to understand in-depth the influence of subretinal transplantation on a broad array of known and unknown functions of RPE. Specifically, we first investigated the genes involved in key functions of RPE (‘visual cycle’, 'phagocytosis’, ‘growth factor’, 'pigmentation’, ‘ECM organization’, ‘oxidoreductase activity,’ and ‘lipid metabolism’). These genes showed progressive increase from ‘early’ to ‘late’ RPE in vitro. Interestingly, in our Tx-RPE cells, we observed that genes involved in ‘ECM organization’, ‘oxidoreductase activity’, and ‘lipid metabolism’ were preferentially increased compared to their in vitro age-matched counterparts. This suggests that these pathway genes may play an integral role in supporting retinal homeostasis, by i) secreting factors for interphotoreceptor matrix formation, ii) protecting against photooxidation through an increased oxidoreductase activity, and iii) actively processing lipid-rich POS ingested into RPE after phagocytosis (30, 46). In contrast, genes involved in these three processes were lowly expressed in vitro, suggesting that these processes were specific to transplanted RPE in vivo, and were triggered by the presence of an overlying photoreceptor layer, possibly light exposure and other unknown factors that may be provided by the in vivo circulation through the choriocapillaris. Collectively, these observations provide in-depth molecular evidence of transplanted RPE adhesion and integration with host photoreceptors in situ within subretinal space, to facilitate retinal homeostasis and maintain retinal structural integrity.

Next, we performed trajectory inference on single-cell data obtained from age-matched in vitro RPE cultures and posttransplanted RPE, to track and understand how specific subpopulations of in vitro RPE may have evolved differently posttransplantation in the subretinal microenvironment. Both in vitro RPE and Tx-RPE demonstrated a similar maturity continuum within their condition. Surprisingly, we observed interconnectivity of corresponding clusters between in vitro and Tx-RPE, suggesting that majority of the in vitro clusters would have survived in vivo regardless of maturity status. This is with the exception of the ‘terminal’ in vitro iPSC-RPE state (Cluster ‘5’), which diverged significantly from the ‘terminal’ Tx-iPSC-RPE state (Cluster ‘6’). We ascertained that out of these two clusters, only Tx-iPSC-RPE matured in the trajectory toward native adult human RPE (Ref_RPE), affirming the favorable directionality of RPE maturation upon transplantation. However, trajectory interference is based on snapshot measurements, and hence should be interpreted as a statistical expectation, rather than actual transition path of the cells. Future studies that include the use of alternative Live-seq technology (47), which allows sequential molecular profiling of the same cell, will be helpful for uncovering actual cell fate changes after transplantation.

To gain deeper insights into this favorable directionality of Tx-RPE cells (for both ESC and iPSC-RPE) toward Ref_RPE, we performed SCENIC analysis and identified five unique overlapping transcription factors (*MAFF*, *JUN*, *FOS*, *RARG,* and *NFIL3*) that were highly enriched specifically in the terminal, most ‘mature’ transplanted RPE subpopulation. These transcription factors further shed light into RPE biology and functions that are acquired only upon transplantation but are not observed in the in vitro matured RPE. We report a broad network of pathways controlled by three key transcription factors, *MAFF*, *JUND,* and *FOS*, which are postulated to play an important role in regulating RPE functions related to photoreceptor maintenance and cell survival based on its downstream targets. These transcription factors are likely to be activated in vivo due to continuous exposure to oxidative stress, arising from the combined effects of exposure to ambient light and choroidal oxygen, daily phagocytosis, and phototransduction that lead to production of high amounts of mitochondrial reactive oxygen species during normal RPE metabolism in the eye (48, 49). These environmental and cellular stressors, present in normal eyes and amplified in diseased eyes, may induce upregulation of prosurvival genes in RPE as means to adapt to the diverse and challenging retinal microenvironment, concurrently demonstrating its ability to tolerate stress. Importantly, these prosurvival factors were observed to be specifically enriched in the terminal population of fully matured transplanted RPE. We postulate that the transplantation of a heterogeneous RPE population into diseased eyes may enrich for this subpopulation, and thus confer a longer term survival advantage. By unravelling the role of this tripartite factors in RPE transplant biology, we propose that the ability of RPE to elicit a robust defense mechanism against the aforementioned stressors would be a key criterion to test pretransplantation to ensure good survivability in vivo. This might include RPE cells with the ability to activate *FOS*, *JUND,* and *MAFF*. Indeed, polarized, and mature RPE monolayer culture in vitro has already been reported to have higher resistance to oxidative stress compared to nonpolarized cells (49, 50). It is possible that this feature was retained in vivo after RPE monolayer transplantation, compared to cell suspension transplantation. Consistent with this, we observed activated regulons of these tripartite transcription factors in the most ‘mature’ state of Tx-RPE cells, that were also active in adult human RPE (Ref_RPE). We hypothesize that in a diseased eye, these subpopulation of RPE may outperform due to its role in maintaining RPE functions and promoting expression of prosurvival factors. Therefore, inducing selective expression of these transcription factors prior to transplantation may improve survivability, function, and host integration, thereby improving efficacy.

In summary, we dissected the transcriptomic landscape of stem cell–derived RPE after subretinal transplantation and deciphered the trajectory of these transplanted cells within the retinal microenvironment. Our findings suggest the positive influence of subretinal transplantation of RPE monolayer, whereby Tx-RPE cells converge toward a mature, adult human RPE state, despite initial heterogeneity in vitro. Furthermore, Tx-RPE have unique GRNs related to resistance toward oxidative stress and RPE cell survival alongside genes supporting specific RPE functions to continually maintain retinal homeostasis. These findings shed insights into unique single-cell transcriptomic changes in RPE that occur in vivo posttransplantation and provides opportunities for therapeutic modulation of these pathways to enhance eventual clinical outcomes for retinal cell therapy.

## Materials and Methods

### Stem Cell Culture.

Human ESC line, H9 (WA09) was a gift from Timothy A. Blenskinsop. Human iPSC line was derived from dermal fibroblasts (HDFa, Thermo Fisher Scientific) and reprogrammed using a modification of EBNA-1 based episomal reprogramming method (51). Both ESCs and iPSCs were maintained in mTeSR^®^ medium (Stem Cell Technologies), on plates precoated with Matrigel^®^ (Corning) and passaged every 5 to 7 d upon reaching 80 to 90% confluency using 0.5 mM EDTA (Sigma).

### Directed RPE Differentiation.

The ESCs and iPSCs were seeded in a 6-well culture dish coated with Matrigel^®^ and grown to 90% confluency before starting the directed differentiation using a modified published protocol (16, 17). At the end of the differentiation, at day 16, the medium was switched to RPE maintenance media (termed RPE medium) (52, 53) containing Dulbecco’s Modified Eagle Medium:Nutrient Mix F-12 (F12, Gibco), supplemented with 2% heat-inactivated fetal bovine serum (Gibco), 1× GlutaMAX (Gibco), 1× MEM nonessential amino acids solution (Gibco), 1× sodium pyruvate (100 mM, Gibco), 1× penicillin-streptomycin (10,000 U/mL, Gibco) and 10 mM nicotinamide (Sigma-Aldrich) and cells were grown to day 30 before enriching them further by purification (54) and replating them onto Transwell inserts (0.4 µm, Corning) precoated with 10 µg/mL synthemax-II (Corning) in RPE medium. The RPE cells were further grown for 30 d before proceeding with characterization on day 60 differentiated cells. The culture was maintained at 37 °C under 5% CO_2_ and medium was changed every 2 to 3 d. Day 60 ESC-RPE (*n *= 4) and iPSC-RPE (*n *= 4) from identical differentiation batches were either further grown for 30 d in vitro (*n *= 1 for each line) or transplanted subretinally into rabbit eyes (*n* *=* 3 for each line) and followed-up for 30 d.

### Reverse Transcription Real-Time (qRT-PCR).

Total RNA from day 60 ESC-RPE (*n *= 3) and iPSC-RPE (*n *= 3) cultures were extracted and purified with the RNeasy Mini Kit (Qiagen) following the manufacturer’s recommended protocol. Then, 1 μg purified RNA was reverse transcribed to cDNA using the iScript cDNA Synthesis Kit (Bio-Rad). This was followed by qPCR with gene-specific primers using the KAPA SYBR FAST qPCR Master Mix (2×) kit (Sigma-Aldrich) on Quantstudio five real-time PCR system (Thermo Fisher Scientific). qPCR experiments were performed in technical triplicates. Data analysis was performed using the comparative CT method. The expression levels were normalized to those of the housekeeping gene, *GAPDH*. The forward (5′ to 3′) and reverse (5′ to 3′) primers used in this study are as follows: *OCT4*, Forward (F): CAGTGCCCGAAACCCACAC and Reverse (R): GGAGACCCAGCAGCCTCAAA; *PMEL17*, F: GTTGATGGCTGTGGTCCTTG and R: CAGTGACTGCTGCTATGTGG; *TYRP2*, F: CTCAGACCAACTTGGCTACAGCTA and R: CAGCACAAAAAGACCAACCAAA; *BEST1*, F: CCTGCTGAACGAGATGAACA and R: CCACAGTCACCACCTGTGTA; *RPE65*, F: CCTGATTCATACCCATCAGAACCC and R: CACCACACTCAGAACTACACCATC; *GAPDH*, F: CAGCCTCAAGATCATCAGCA and R: TGTGGTCATGAGTCCTTCCA.

### Immunofluorescence (IF) of In Vitro ESC- and iPSC-RPE Cultures.

Day 60 ESC-RPE (*n *= 3) and iPSC-RPE (*n *= 3) cultures grown on Transwell inserts (Corning) were fixed with 4% paraformaldehyde (pH 7.4) for 20 min at room temperature (RT), permeabilized with 0.2% Triton X-100 for 5 min and blocked with 1% bovine serum albumin (BSA, Sigma-Aldrich) in phosphate-buffered saline (PBS) for 1 h. After removing blocking buffer, cells were incubated with primary antibodies diluted in 1% BSA in PBS overnight at 4 °C. After three washes to remove the primary antibodies, cells were incubated with respective secondary antibodies (Alexa Fluor 488, 1:1000, Thermo Fisher Scientific) against its host species and Hoechst 33,342 solution (1:1000, Thermo Fisher Scientific) for 30 min at RT. After three washes to remove the secondary antibodies, cells were mounted using Fluorsave (Millipore) and imaged using LSM700 confocal microscopy (Zeiss). The primary antibodies used were Zonula Occludens-1 (ZO-1, 1:100, Thermo Fisher Scientific, 617300), RPE65 (1:125, Abcam, ab13826), BEST1 (1:100, Abcam, ab2182) and Na^+^/K^+^ ATPase (1:100, Thermo Fisher Scientific, MA3-915).

### TEER.

Day 30 ESC-RPE (*n *= 3) and iPSC-RPE (*n *= 3) after replating onto Transwell inserts (Corning) were monitored using TEER probes weekly for another 8 wk (day 30 to 86) using the Epithelial Volt Ohm meter (EVOM2, World Precision Instruments) following the manufacturer’s instructions. Net TEER (Ohms·cm^2^) was calculated by subtracting the resistance (Ohms) values of experimental Transwells containing RPE from those of blank Transwells serving as no cell controls and multiplying net values by the area of the insert (0.33 cm^2^).

### POS Phagocytosis Assay.

POS were isolated from porcine eyes (obtained from Abbatoir) and labeled with fluorescein isothiocyanate (FITC, Thermo Fisher Scientific) as described previously (55). For phagocytosis assay, the day 60 ESC- (*n *= 3) and iPSC-RPE (*n *= 3) on Transwell inserts were challenged with FITC-labeled POS for 2 h at 37 °C and 4 °C. The cells were then washed with PBS thrice, dissociated with TrypLE Express (Thermo Fisher Scientific). Single cells were then subjected to flow cytometry (BS LSR II Flow Cytometer) to determine the percentage of RPE cells positive for FITC fluorescence (internalized FITC-POS).

### Enzyme-Linked Immunosorbent Assay (ELISA) for PEDF and VEGF.

Day 60 ESC-RPE (*n *= 3) and iPSC-RPE (*n *= 3) cultures grown on Transwell inserts were used to perform ELISA for VEGF and PEDF. Medium was collected from the inner chamber (referred to as apical) and outer chamber (referred to as basal) of Transwell inserts, 48 h after complete medium change. The concentrations of growth factors VEGF or PEDF were determined by sandwich ELISA using the VEGF Human ELISA Kit (Thermo Fisher Scientific) or the PEDF ELISA Kit (ChemiKine) according to the manufacturer’s instructions, respectively. Optical densities for both VEGF and PEDF were obtained using a microplate reader (Tecan) at 450-nm wavelength.

### Statistical Analyses.

All in vitro data were reported as mean ± SD. Data normality were assumed for all sample sets. Statistical significance between two groups was determined using Student’s unpaired *t* test. For comparison between more than two groups, one-way (ANOVA) followed by pairwise testing with Tukey’s honest significance difference (HSD) post hoc test was performed. *P* values below 0.05 were considered significant. All analyses were performed using GraphPad Prism (ver. 8.1.1). Sample sizes for each experiment are presented in the corresponding figure caption.

### Animals.

Six adult Dutch belted rabbits (body weight: 1.5 to 2.0 kg) were obtained from Covance (Covance Research Products Inc.). All animal experiments were designed and conducted in accordance with the Association for Research in Vision and Ophthalmology (ARVO) Statement for the Use of Animals in Ophthalmic and Vision Research and approved by the Institutional Animal Care and Use Committee of the SingHealth Experimental Medicine Center (Singapore). All animal procedures were performed in American Animal Association LAC International approved facility.

### Subretinal Transplantation Surgery.

The surgical procedure was carried as published protocols (56, 57). In brief, prior to the surgery, rabbits were anesthetized by intramuscular injection of 50 mg/kg ketamine and 10 mg/kg xylazine and pupils dilated with 2.5% phenylephrine and 1% tropicamide eye drops. Standard 25-gauge (G) vitreoretinal surgery was performed using chandelier illumination, followed by partial surgical removal of the vitreous at the posterior pole in rabbits. Triamcinolone was used to visualize the induction of posterior vitreous detachment. A small bleb retinal detachment was gently raised by manual subretinal injection of balanced salt solution via a 38G cannula (MedOne Surgical) with intraocular pressure temporally set at 4 mmHg (20). The retinotomy was enlarged with vertical vitreous scissors (Geuder). Preloaded bullet-shaped day 60 RPE monolayer implants (ESC-RPE, *n *= 3; iPSC-RPE, *n *= 3) were delivered subretinally using a custom-made device (45, 58). All three transplantation surgeries for each RPE line were performed on the same day to ensure adherence to an exact 30-d period in the eye. RPE implants were placed with the cell monolayer facing the host photoreceptors. Microscope-integrated intraoperative OCT was used to confirm the graft position. A normal intraocular pressure was confirmed before the sclerotomies were sutured with 7-0 vicryl sutures. A topical antibiotic and steroid ointment (Tobradex, tobramycin, and dexamethasone, Alcon) were applied to the treated eyes twice a day for 5 d postsurgery. Only one eye per rabbit was used for the surgical procedure, and one RPE implant was placed per eye. The contralateral eye of the rabbits served as nonoperated controls.

### In Vivo Ophthalmic Follow-Up and End Point Procedure.

Noninvasive in vivo ophthalmic multimodal imaging follow-up were carried out at 1, 2, and 4 weeks postsurgery. Color FP was taken by a biomicroscope (TRC-50DX, TopCon Corp.) Infrared fundus and OCT were performed using Heidelberg Spectralis^®^ HRA + OCT (Heidelberg Engineering). All imaging procedures were done in sedated animals, and pupils were dilated before every imaging as described above. Full-field ERG was carried out to assess retinal function at 30 d postsurgery, using an Espion system (Diagnosis LLC) with protocols and procedures based upon those recommended for humans by the International Society for Clinical Electrophysiology of Vision (59). Animals were dark-adapted at least 20 min before assessment and sedated, and pupils were dilated as described previously. The individual components of an ERG recording were named according to the adaptive state of the eye (DA: dark-adapted; LA: light-adapted) and the stimulus strength. Thus, DA 0.01 reflects the use of a 0.01 cd.s.m^−2^ flash delivered under dark adaptation. At the end point (i.e., 1 mo postsurgery), rabbits were sacrificed in deep anesthesia with an intracardiac injection of the euthanizing agent (Pentobarbital). Following perfusion via the carotid artery with 10% neutral buffered formalin (Leica Biosystem) (57), the eyes were enucleated before proceeding with either IF of the rabbit retina or single-cell dissociation for scRNA-seq.

#### IF of Transplanted RPE in Rabbit Retina Sections.

Following enucleation of a rabbit eye, the entire globe was immersed in 10% formalin for 48 h at 4 °C. The whole eye was then embedded in paraffin. Sections were cut at 10-µm thickness with a microtome (Leica Biosystem) before proceeding with IF. The paraffin-embedded sections were deparaffinized in xylene followed by rehydration through a series of graded ethanol concentrations. The sections were then subjected to antigen retrieval. After which, they were blocked in 10% animal serum in PBS with 0.1% Tween-20 and 1% BSA (Sigma-Aldrich) for 1 h at RT and incubated with primary antibodies overnight at 4 °C. The following primary antibodies were used, anti-TRA-1-85 (1:50, BD Biosciences, 563020), anti-Orthodenticle Homeobox 2 (OTX2, 1:100, Abcam, ab92326), anti-RPE65 (1:125, Abcam, ab13826), and anti-c-Fos (1:500, Santa Cruz Biotechnology, sc-52). After three washes to remove the primary antibodies, the sections were incubated with respective Alexa Fluor secondary antibodies (against primary antibody’s host species) diluted in PBS with 0.1% Tween-20 were added for 30 min at RT. A secondary antibody only negative control was included where sections were only incubated with blocking buffer in place of primary antibodies. Nuclei were stained with Hoechst 33,342 (Thermo Fisher Scientific). Sections were mounted using ProLong Gold Antifade (Thermo Fisher Scientific) and imaged using a LSM8000 confocal laser microscope (Zeiss).

### Sample Dissociation for scRNA-seq.

For in vitro RPE cultures, day 90 ESC-RPE (*n *= 1) and iPSC-RPE (*n *= 1) grown on Transwell inserts were dissociated into single cells with TrypLE Express for 30 min, at 37 °C, 5% CO_2_ and resuspended to 1,000 cells/μL in 0.04% BSA in PBS prior to scRNA-seq. For transplanted RPE (Tx-RPE), six animals (Tx-ESC-RPE: *n *= 3; Tx-iPSC-RPE: *n *= 3) were sacrificed in deep anesthesia at 4 wk postsurgery (i.e., cumulative age of RPE = day 90). After enucleation, the entire globes were washed thoroughly with PBS. The anterior segments were removed, and posterior poles (approximately 5 × 5 mm) were cut from the globes. The neuroretina was carefully removed, and the implant was taken out, and transferred into an Eppendorf tube with 500 μL PBS, using intraocular forceps (Alcon). The cells on implant were dissociated with 0.25% Trypsin-EDTA (Gibco) for 20 to 30 min at 37 °C, 5% CO_2_ with gentle resuspending every 5 min (25). Tx-RPE cells were filtered using a 30-µm strainer and resuspended in 0.04% BSA in PBS. RPE cell counting was performed. Due to the low number of Tx-RPE cells retrieved, we spiked-in them with A549 (CCL-185, ATCC) immortalized cells to avoid library preparation failure due to insufficient cell count. The concentration was adjusted to 1,000 cells/μL in 0.04% BSA in PBS prior to scRNA-seq.

### ScRNA-seq Library Preparation.

Single-cell suspensions from in vitro ESC- and iPSC-RPE (*n *= 1 per line) and from Tx-ESC- and Tx-iPSC-RPE (*n *= 3 per line) were maintained at 4 °C for less than 30 min before being transported to the 10× Genomics Chromium instrument for scRNA-seq. Using the 10× Genomics Single Cell 3′ Reagent Dual Index Kit V3, we performed droplet-based single-cell encapsulation, followed by bar-coded reverse transcription to generate the bar-coded complementary DNA (cDNA) library. The eight cDNA libraries were sent for high-throughput DNA Sequencing at the Genome Institute of Singapore, Next Generation Sequencing Facility (IGP-NGSP, A*STAR, Singapore).

### High-throughput DNA Sequencing and Data Processing.

The eight cDNA libraries were amplified and ligated with Illumina adaptor sequences, adjusted to appropriate concentration, pooled, and sent for high-throughput DNA sequencing on Illumina NovaSeq 6000. Each pool of eight samples were ran on three individual lanes to minimize technical variability. The sequencing library format chosen was 150-bp pair-end. Software from Illumina was used to output the sequencing data in FASTQ format. In total, 11569355486 reads were obtained from eight samples. This is on average 1446169436 reads per sample. This sequencing depth is similar to other published 10× Genomics scRNA-seq libraries, and the saturation curve (*SI Appendix*, Fig. S6*A*) indicates that this read depth was sufficient to achieve optimal dynamic range and deep enough to provide robust gene detection for each cell. The density plots of read counts and gene counts per cell (*SI Appendix*, Fig. S6 *B* and *C*) show a relatively consistent distribution across samples, comparable to other scRNA-seq datasets, and show sufficient number of genes are detected for each cell. In addition, we also plotted the percentages of reads that fall onto the mitochondrial genome, in order to ensure that the cells are healthy and maintaining low read proportion of reads mapped to mitochondrial DNA (*SI Appendix*, Fig. S6*D*). Further quality control (QC) checks were performed using FASTQC (*SI Appendix*, Figs. S3 and S4), which is important to verify that all sequencing libraries were of high quality and important attributes such as sequence quality score distributions were within expected ranges. Because the QC attributes fell within the normal acceptable range across samples, we decided batch correction was not necessary.

### ScRNA-seq Data Analyses.

#### Read mapping.

Cell ranger 4.0.0 (60) was used to map the sequencing reads in the FASTQ files onto a custom reference genome we prepared. The custom reference genome contains both the human (GRCh38) and the rabbit (OryCun2.0) reference genome sequences being concatenated together. The reason that both references were required was because there was some contamination from surrounding rabbit cells during harvest of transplanted human stem cell–derived RPE cells. The reason that both references were concatenated for mapping together rather than mapping to one reference genome after another reference genome, was that mapping to the concatenated genome enabled the fair competition of each FASTQ read onto both genomes, thus giving the most accurate estimation of the percentage of DNA reads originating from human or originating from rabbit. Only cells with more than 95% DNA reads mapped to human reference are considered as human stem cell–derived RPE cells (*SI Appendix*, Fig. S5). The remainder cells (<5%) were considered as rabbit cells or other cells. All our analysis was focused on the human origin RPE cells; thus, we removed nonhuman cells. Subsequently, for all the cells retained for analyses (i.e., marked as human cells), we deleted the very small number of rabbit reads that mapped to the human reference genome due to cross-species homology. The aforementioned procedure generated a matrix with each row representing one human gene and each column representing one cell. The counts are integers representing the number of reads for the corresponding gene in that corresponding cell. The matrices were imported into Seurat 4.1.1 (61) for further scRNA-seq bioinformatics analysis and data visualization.

#### Cell quality filtering.

For filtering of cells, each cell had to satisfy all of the following: i) Minimum number of cells expressing a single gene needs to be more than 3; ii) the percentage of reads that mapped to mitochondrial shall be less than 20%; and iii) the total number of unique genes with reads > 0 needs to be more than 500 genes. In addition, spiked-in A459 cells (CCL-185, ATCC) were filtered-out by removing cells with high expression (log normalization > 1) of *RSPO3*, *GPRIN2* and, *TM4SF20* genes specific to A459 (62). Finally, the cell clusters expressing markers specific to non-RPE cell types (*SI Appendix*, Table S1) were also removed, leaving only the RPE and committed RPE for further analysis. A total of 13,232 individual RPE cells (both ESC- and iPSC-derived) passed the above-mentioned quality filter (*SI Appendix*, Table S2). In vitro RPE: 10,772 cells and Tx-RPE: 2,460 cells. Density distribution plots showing the total number of mapped reads per cell, total number of genes having >1 mapped read per cell and percentage of total genes mapped to mitochondrial genes, as well as the saturation curve were plotted using the dittoSeq R package v. 1.10 (https://github.com/dtm2451/dittoSeq).

#### Evaluating stably expressed genes between samples.

To assess whether there were any confounding sources of technical variation between samples, the expression of three sets of stably expressed (i.e., housekeeping) genes from the gene ontology were evaluated. Genes involved in tRNA aminoacylation (GO:0043039), spliceosome assembly (GO:0005681), and protein translation initiation factors (GO:0006413) were used to generate density distribution plots showing the percentage of total reads mapping to each of these gene sets across samples (*SI Appendix*, Fig. S6*E*). Since the density distribution plots of housekeeping genes did not show significant alteration between the in vitro and posttransplanted samples, no batch correction between them was performed.

#### Normalization, dimensionality reduction, and UMAP visualization.

The count matrix of all 13232 RPE cells was normalized using “sctransform” (63) within the Seurat 4.1.1. package. Dimension reduction by principal components analysis (PCA) was performed on the normalized data, and the principal components were used for subsequent analyses, including UMAP visualization and unsupervised clustering analyses. In order to visualize the relative distances between cells and cell clusters on 2D, while preserving information from higher dimensions, we chose to use UMAP (26) to project the cells.

#### Unsupervised clustering.

Independent of the above-mentioned visualization, unsupervised clustering of cells was performed using the Seurat package on the above-mentioned final matrix. First, the “Approximate Nearest Neighbors Oh Yeah” package with the cosine distance metric was used to compute the k-nearest neighbors (k-NN). Then the Louvain clustering algorithm (64) was used to partition the cells into clusters.

#### Differential expression analysis.

Differential expression analyses between cell subpopulations were performed using DESeq2 (65) on the raw read count matrix, whereby the Wald test was used for hypothesis testing. The Wald statistic was used to rank all genes, and this ordered list was used as input for gene set enrichment analysis (GSEA) (66). GSEA was performed using the R package ‘fgsea’ using a concatenation of all three gene ontology databases (GO:BP, GO:MF, GO:CC) downloaded from MSigDB (http://www.gsea-msigdb.org).

#### Integration and batch correction between external RPE-choroid tissue data and our dataset.

A reference healthy adult human RPE-Choroid scRNA-seq dataset (32) was integrated and batch effect corrected with our samples according to the following procedure described. The expression matrix of healthy macula and peripheral donors were downloaded from GEO database (https://www.ncbi.nlm.nih.gov/geo) (accession no. GSE135922). The expression matrix of downloaded dataset and the expression matrix of our dataset underwent the exact same log normalization process of the aggregated reads using Seurat “NormalizeData” function, whereby read counts for each cell were divided by the total number of reads for that cell, scaled by a factor of 10,000 and then log transformed. Thereafter, the normalized expression matrix of the two datasets were integrated and batch-effect corrected using fast mutual nearest neighbors correction (fastMNN) from the batchelor R package v1.10.0 (67). The top 3,000 most variably expressed genes from each dataset were used during the fastMNN integration and batch correction step. The resulting integrated and batch-effect corrected matrix was used for dimension reduction visualization using UMAP, as well as all subsequent analyses that included the reference dataset.

#### PAGA analysis.

PAGA (33) from the Scanpy toolkit (68) was used for trajectory inference on in vitro and Tx-RPE cells, which is based on estimating connectivity between manifold partitions from a topology preserving map of the cells. PCA was performed using a set of genes known to be involved in RPE function, and UMAP was used to estimate the connectivity between data points, from which a k-NN graph was constructed in the low-dimensional embedding space. Louvain clustering (64) was performed on the k-NN graph to generate the partitions for the trajectory graph. The in-built force-directed graph drawing algorithm was then used to visualize the cells on the low-dimensional embedding and the clustering results from Louvain cluster are shown on the graph as different colors.

#### Construction of GRNs.

PySCENIC (69) was used to construct GRNs and to estimate regulon activity for each cell. The “GRNBoost2” option of algorithm was selected in the ‘grn’ step of PySCENIC, which was run on the count matrix for each cell line separately, together with the adult human reference RPE (Ref_RPE) to derive gene coexpression modules. Subsequently, the ‘ctx’ option was used to find transcription factor-enriched motifs within the target genes. Last, the ‘auc’ option was used to calculate AUC enrichment scores to quantify the activity of regulons across single cells. A binarization algorithm was used to determine if a regulon is active or not based on the bimodal distribution of the AUC scores for that regulon across single cells. These binarized regulon activity scores where then used to compare the Ref_RPE with each RPE line and a UMAP was constructed based on these. Logistic regression was applied to the binarized AUC scores to identify regulons with statistically significant changes in their activity after transplantation and Cytoscape (70) was used to visualize the regulons.

## Supplementary Material

Appendix 01 (PDF)

## Data Availability

All computations were made using Python v3.8.1 and R v4.1.3. Plots were generated using matplotlib v3.3.2, seaborn v0.11.0, and ggplot2 v3.3.6. The authors declare that all data supporting the results in this study are available within the paper and its *SI Appendix*. The scRNA-seq data is available from the Gene Expression Omnibus (GSE), with the dataset identifier GSE212896 (71) [https://www.ncbi.nlm.nih.gov/geo/query/acc.cgi?acc=GSE212896].
