# Supplementary material for: Single-cell transcriptomics reveals maturation of transplanted stem cell–derived retinal pigment epithelial cells toward native state
Source: Proc Natl Acad Sci U S A. 2023 Jun 20;120(26):e2214842120. doi: 10.1073/pnas.2214842120 (PMC10293804; doi:10.1073/pnas.2214842120)
Supplement: Supplementary file 1 — Appendix 01 (PDF) [file pnas.2214842120.sapp.pdf]

PNAS

Supporting Information for

Single-cell transcriptomics reveals maturation of transplanted stem cell-derived retinal pigment epithelial cells towards native state

†Bhav Harshad Parikh[1], †Paul Blakeley[1,2], Regha Kakkad[1,2], Zengping Liu[1,2,3], Binxia Yang[1], Mayuri Bhargava[1,2,4], Daniel Soo Lin Wong[2], Queenie Shu Woon Tan[1], Claudine See Wei Wong[1], Hao Fei Wang[1], Abdurrahmaan Al-Mubaarak[1,2], Chai Chou[5], Chui Ming Gemmy Cheung[3], Kah Leong Lim[5], Veluchamy Amutha Barathi[2,3,6], Walter Hunziker[1,7], Gopal Lingam[2,3,4], Tim Xiaoming Hu*[1], Xinyi Su*[1,2,3,4]

Contact Information of Corresponding Authors

Tim Xiaoming Hu: Email: huxm@imcb.a-star.edu.sg

Xinyi Su: Email: xysu@imcb.a-star.edu.sg

This PDF file includes:

Figures S1 to S11
Tables S1 to S2

Supporting Information Figures

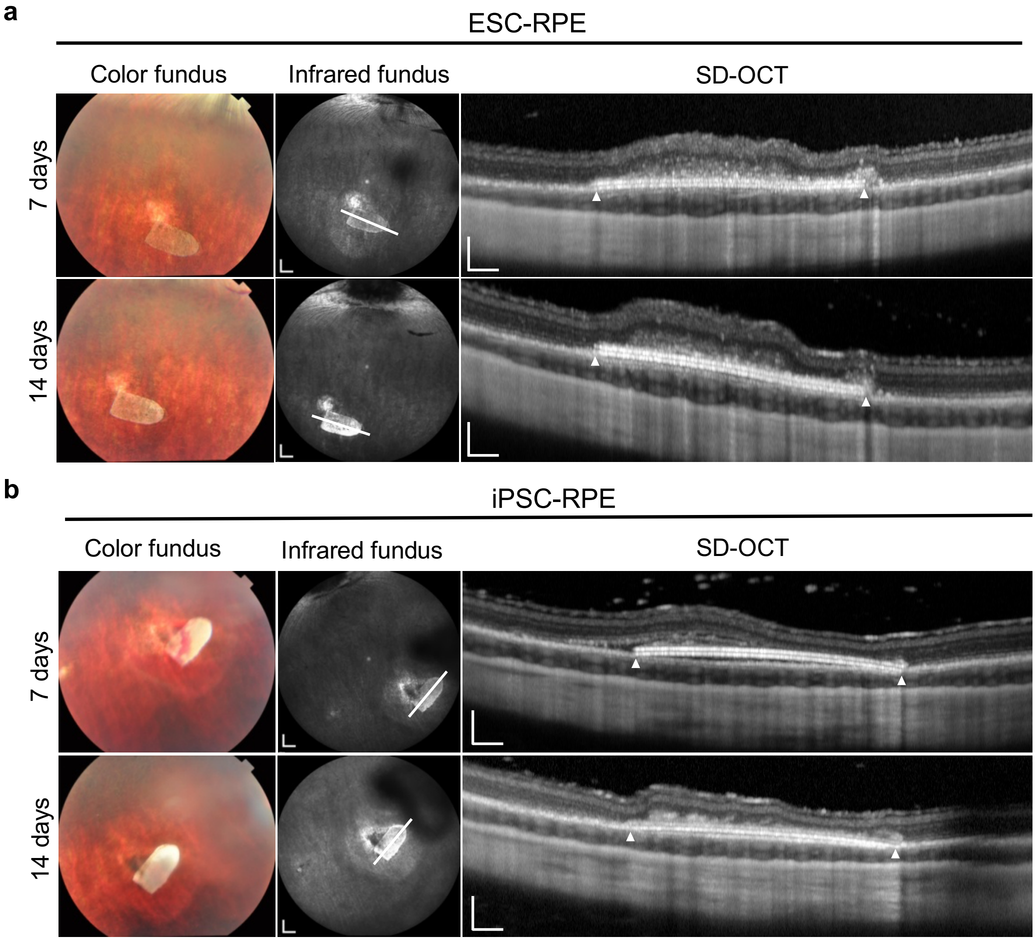

Fig. S1 | Live ophthalmic imaging of rabbit eyes with subretinally transplanted RPE monolayers at early time points. *In vivo* follow-up of subretinally transplanted (**a**) ESC-RPE monolayers (*n*=3 rabbits) and (**b**) iPSC-RPE monolayers (*n*=3 rabbits) at 7 days and 14 days post-surgery with imaging modalities of color fundus, infrared fundus, and spectral domain-optical coherence tomography (SD-OCT). One representative image is shown. The bullet shaped RPE implants were visible in the color fundus images. The white lines indicated positions at which SD-OCT cross-section images were taken. The white triangles indicated the boundaries of the implants on SD-OCT. Scale bar, 2 mm in infrared fundus, 200 μm in SD-OCT images.

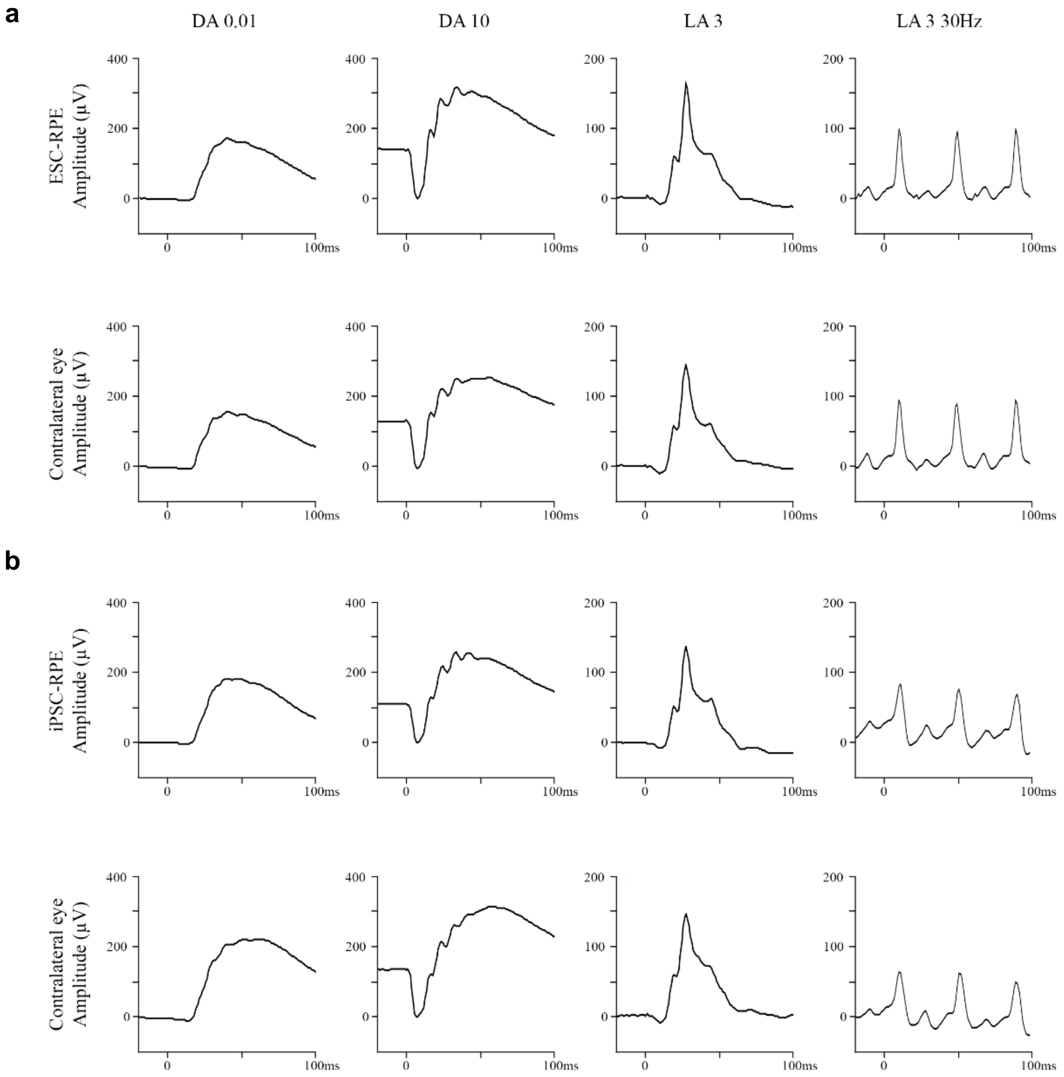

Fig. S2 | Measurement of retinal function using full-field electroretinography (ERG) at 30 days post-transplantation. A representative ERG of rabbits transplanted with (**a**) ESC-RPE (*n*=3) and (**b**) iPSC-RPE (*n*=3) for DA 0.01, DA 10, LA 3 and LA 3 30Hz. The waveforms of contralateral eyes are from the same rabbit, but without any surgical procedure and represent the healthy eye. All waveforms from the transplanted eye remained normal. DA = dark adapted. LA = light adapted.

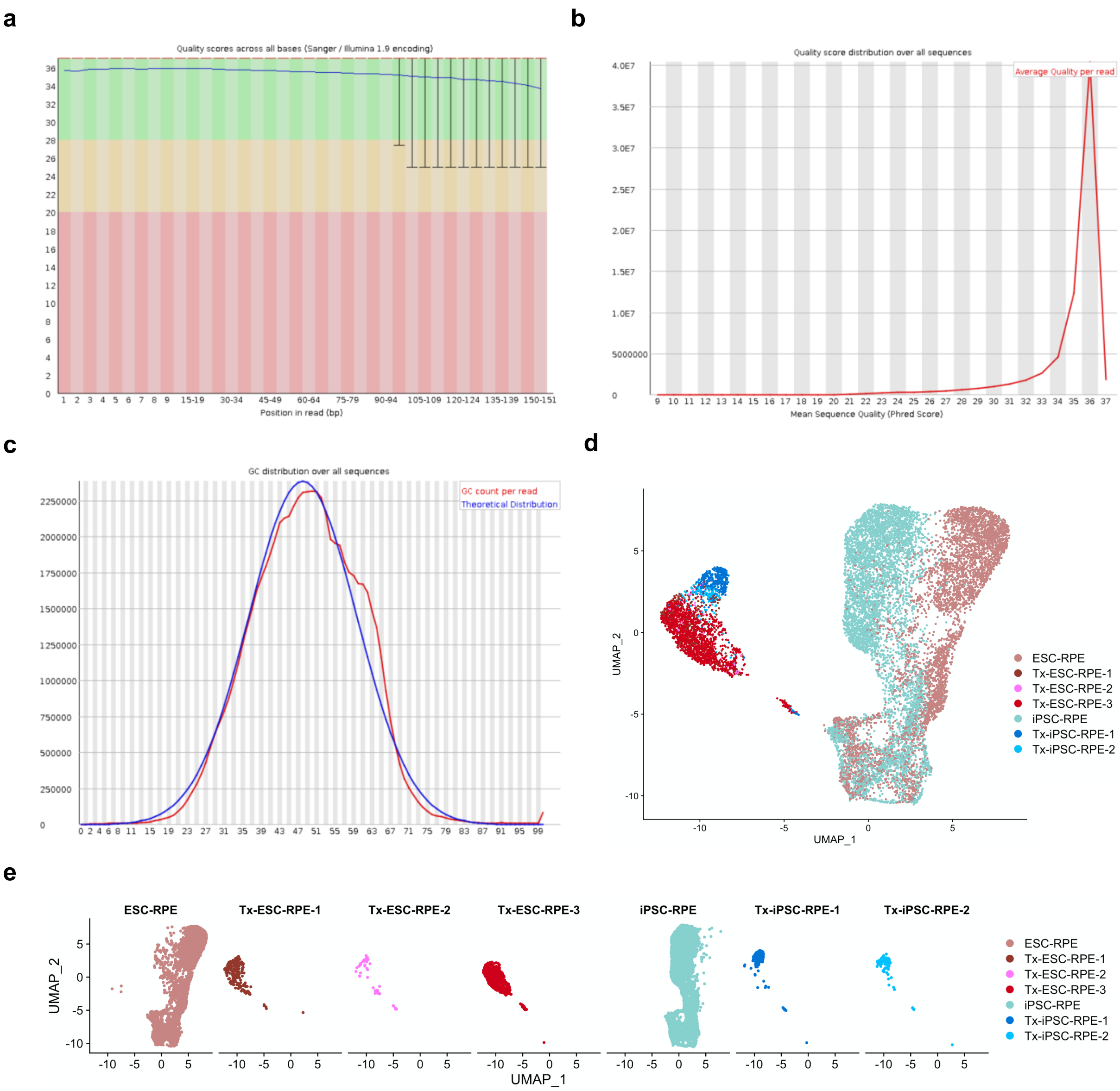

Fig. S3 | Quality control analyses using FASTQC and UMAP. (**a**) The FASTQC software was run on all samples and the representative fastqc result is shown. The sequencing quality scores graph showing high base-pair quality was maintained over every base-pair position across entire read length. (**b**) Quality score distribution plot showed a clear uni-modal distribution around Score 35-37, which is almost the highest range of scores achievable. (**c**) The theoretical distribution of GC ratio assuming no GC bias, is shown as the blue line. The actual GC ratio distribution across all sequenced reads is shown in red line. The red line closely matches the theoretical distribution, indicating GC bias of our experiment is negligible. (**d**) The UMAP visualization of all cells in the experiment. Different biological replicates and different samples are indicated by different colors. The biological replicates of the same condition are positioned closely to each other, suggesting that the any technical and/or biological variations during the experiment are minimal, relative to the effect caused by transplantation. (**e**) The same UMAP as in (d), was also visualized using the “split option” such that each sub-plot is showing the distribution of individual samples.

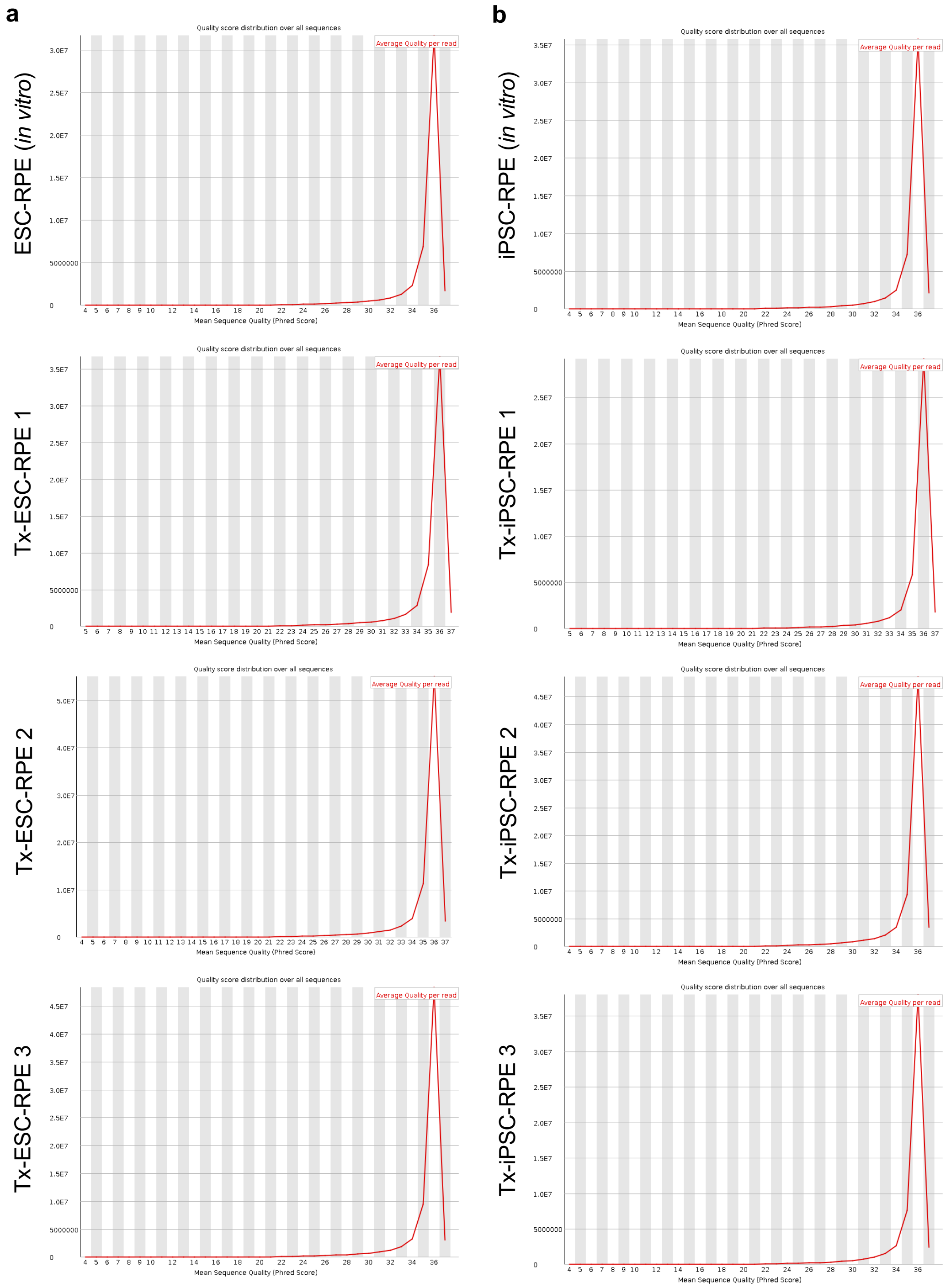

Fig. S4 | Per sequence quality score distributions calculated using FASTQC. (a) ESC-RPE (*in vitro*, *n*=1) and Tx-ESC-RPE (*n*=3). (**b**) iPSC-RPE (*in vitro*, *n*=1) and Tx-iPSC-RPE (*n*=3). Most of the reads contained in the FASTQ files have high average sequence quality across all samples.

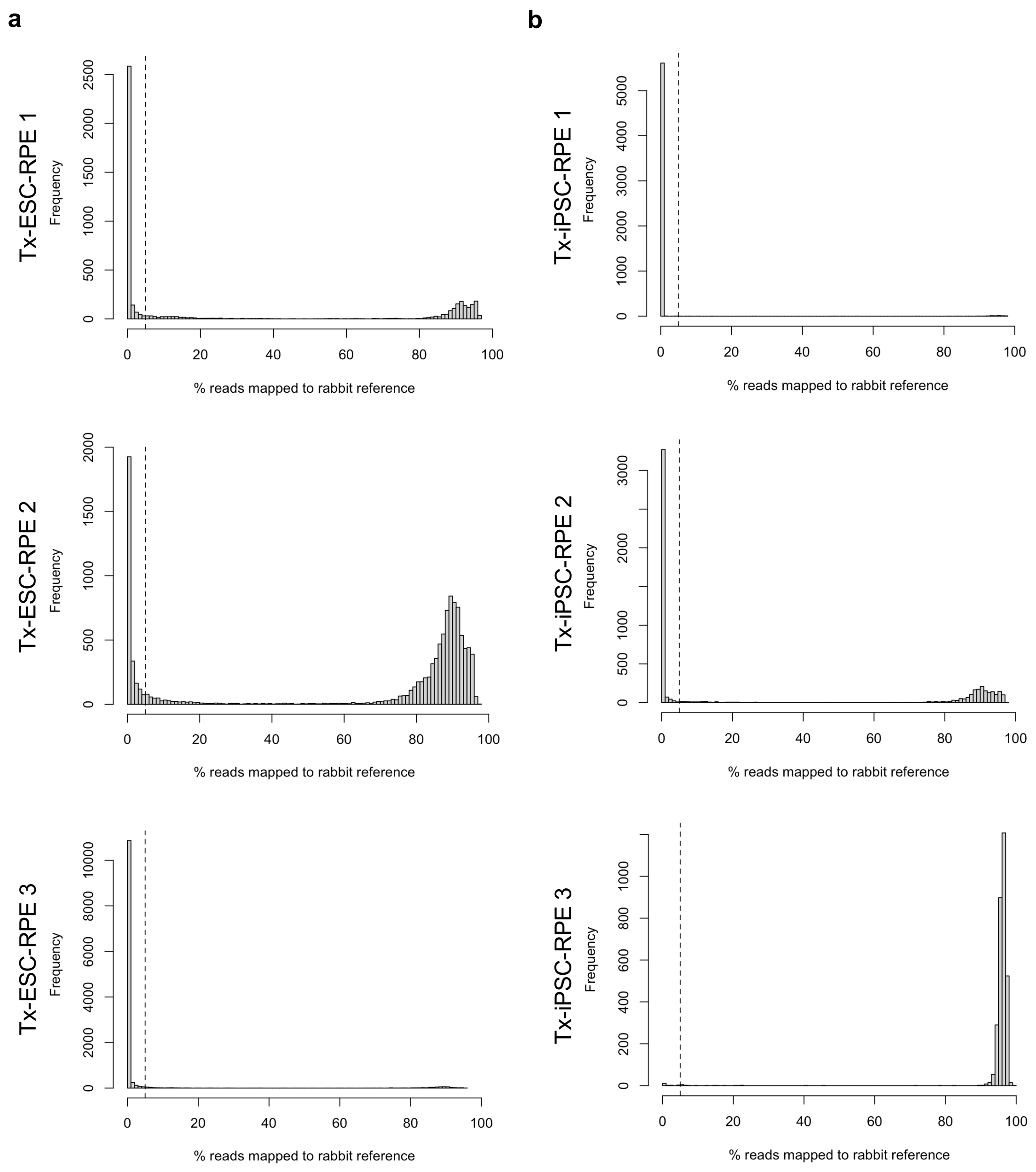

Fig. S5 | Mapping to rabbit reference genome. Density distribution of cells from (**a**) Tx-ESC-RPE (*n*=3) and (**b**) Tx-iPSC-RPE (*n*=3) showing percentage of reads mapping to the rabbit reference genome. Vertical line indicates 5% threshold for retaining cells for downstream analysis.

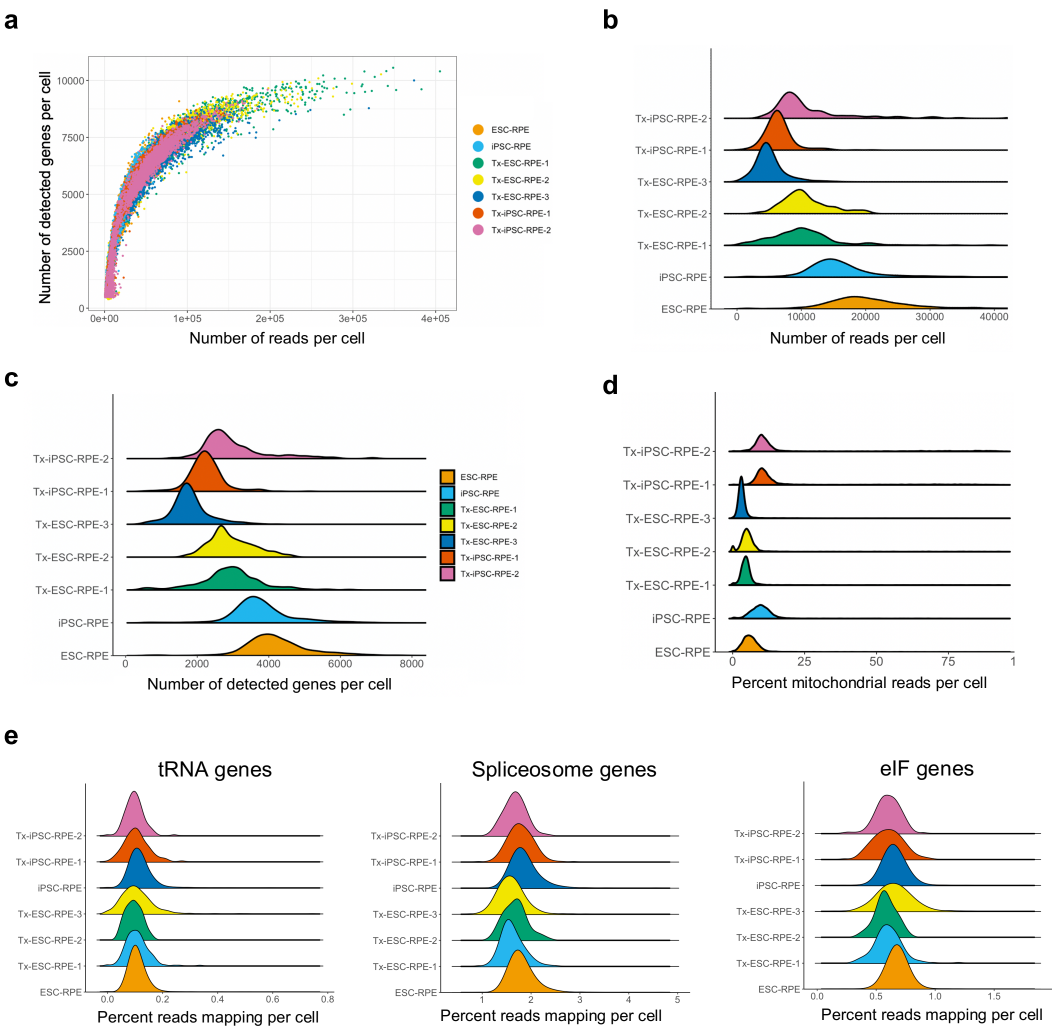

Fig. S6 | Total number of reads and genes per cell after cell quality filtering. (**a**) Scatter plot of number of reads and number of expressed genes. Density distribution plots were generated for all 7 samples that had passed the cell quality filtering and shows the (**b**) distribution of the number of sequencing reads per cell, (**c**) number of expressed genes per cell (genes having at least 1 mapped read in that cell are defined as expressed), (**d**) percentage of reads mapping to the mitochondrial genome. (**e**) Density distribution plot was also generated to show the fraction of reads, in each cell, that are mapped to tRNA aminoacylation genes, spliceosome assembly genes, and protein translation initiation factors (eIF) genes. The sample-to-sample variation is negligible indicating the absence of batch effect.

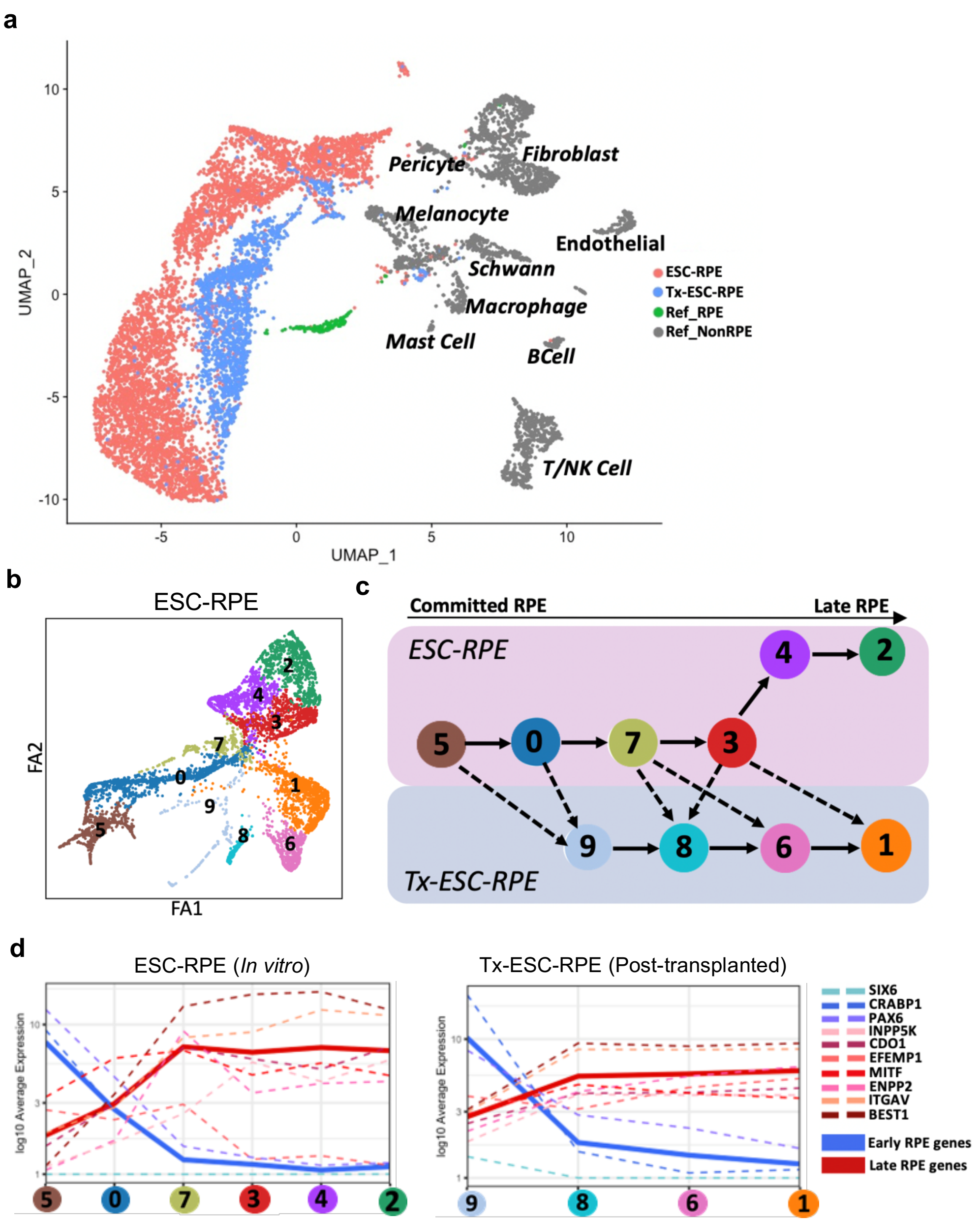

Fig. S7 | UMAP, trajectory, and marker gene expression analyses of ESC-RPE. (**a**) All ESC-RPE cells were plotted together with a reference panel of different cell types from healthy adult human RPE-choroid tissue. Transplanted ESC-RPE (Tx-ESC-RPE), followed by *in vitro* ESC-RPE, were closest to the reference RPE (Ref_RPE). (**b**) Force-directed graph showing the trajectory of ESC-RPE subpopulations before and after transplantation. (**c**) PAGA analyses generated a connectivity graph, showing the pathway of maturation for different clusters as defined in (b). The solid arrows indicate the connectivity graph within the same condition. The direction of the dashed arrows was inferred based on the maturation trajectory. (**d**) Using the clusters obtained in (b), the average expression of selected RPE-specific genes within a cluster were plotted as a dashed curve onto the maturation trajectory. The thick solid curve is the average of 'early' RPE markers (blue) and 'late' RPE markers (red). These analyses were repeated for both *in vitro* and post-transplantation ESC-RPE.

a

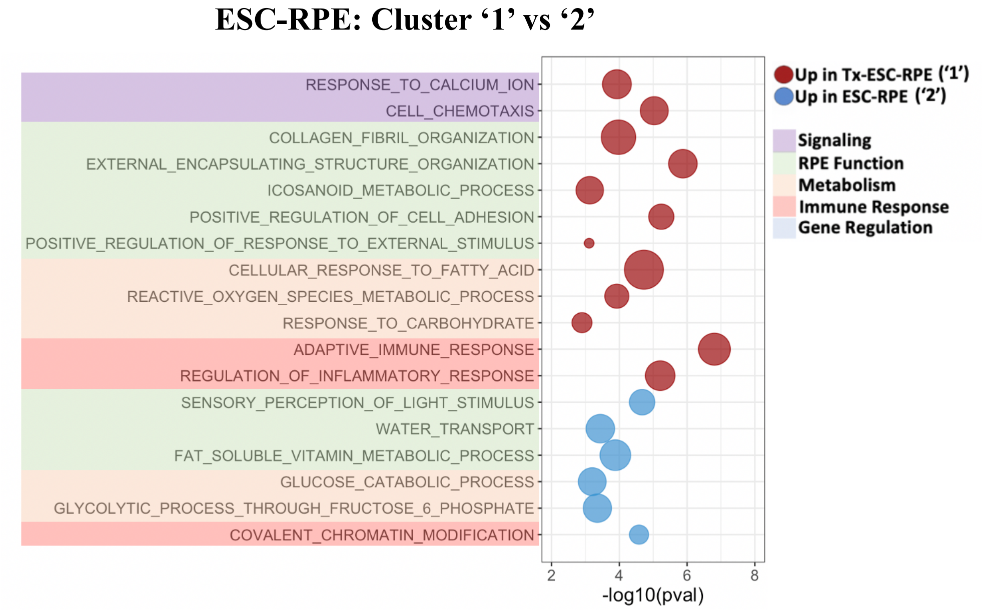

b

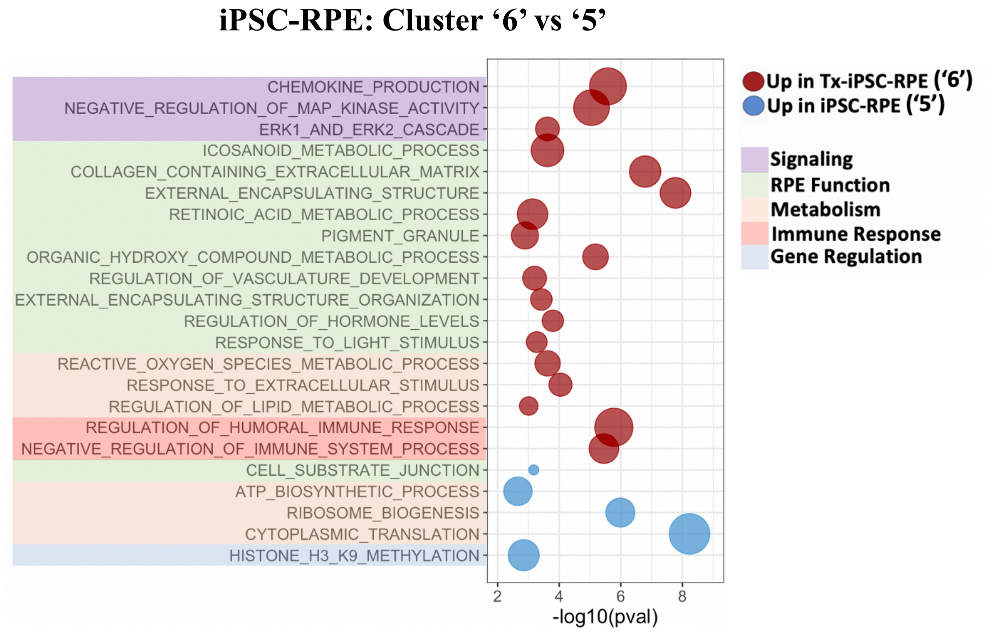

Fig. S8 | Pathway analyses showing differences between 'late' clusters of *vitro* and post-transplanted RPE lines. (**a**) For ESC-RPE, based on the results in *Fig. S4b* and *c*, cluster '1' and cluster '2' were compared to show their upregulated pathways. (**b**) Same analysis as (a) except that this analysis was performed on iPSC-RPE, based on the clustering results shown in *Fig. 3C* and *D,* cluster '6' and cluster '5'. The enriched pathways were categorized into 'Signaling', 'RPE Function', 'Metabolism', 'Immune Response' and 'Gene Regulation' and shown as up in either condition (post-transplanted or *in vitro*).

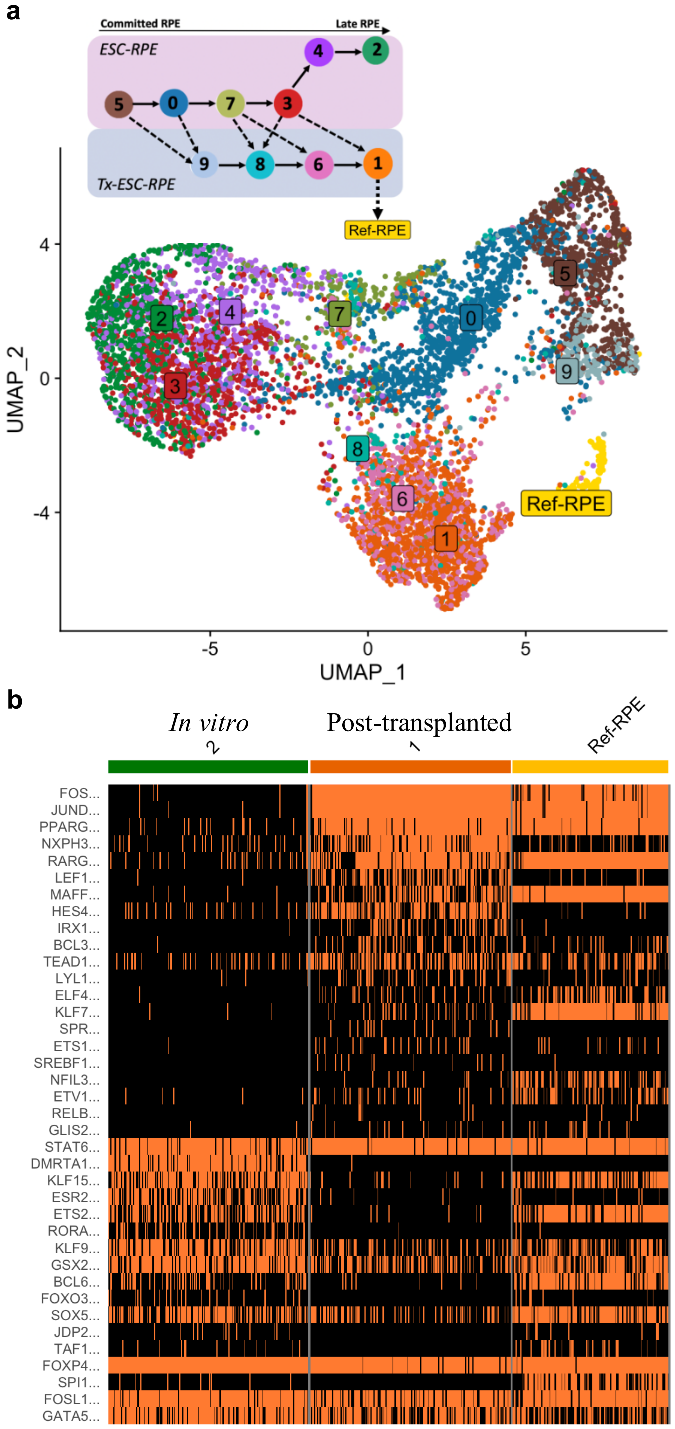

Fig. S9 | Regulon analyses using SCENIC shows that post-transplantation, 'late' Tx-ESC-RPE are highly similar to human reference RPE. (**a**) UMAP dot plot based on binarized AUC scores. 'Late' Tx-ESC-RPE (cluster '1') is very close to Ref-RPE, suggesting high similarity to adult human RPE cells in their regulon status. The dots are colored by PAGA trajectory states based on *Fig. S4b*. (**b**) Heatmap showing regulons with elevated activity (Likelihood Ratio Test: $P_{adj} < 0.05$) between Tx-ESC-RPE (cluster '1') and ESC-RPE (cluster '2'), based on binarized AUC scores. The regulon activity heatmap of Ref-RPE is shown on the side.

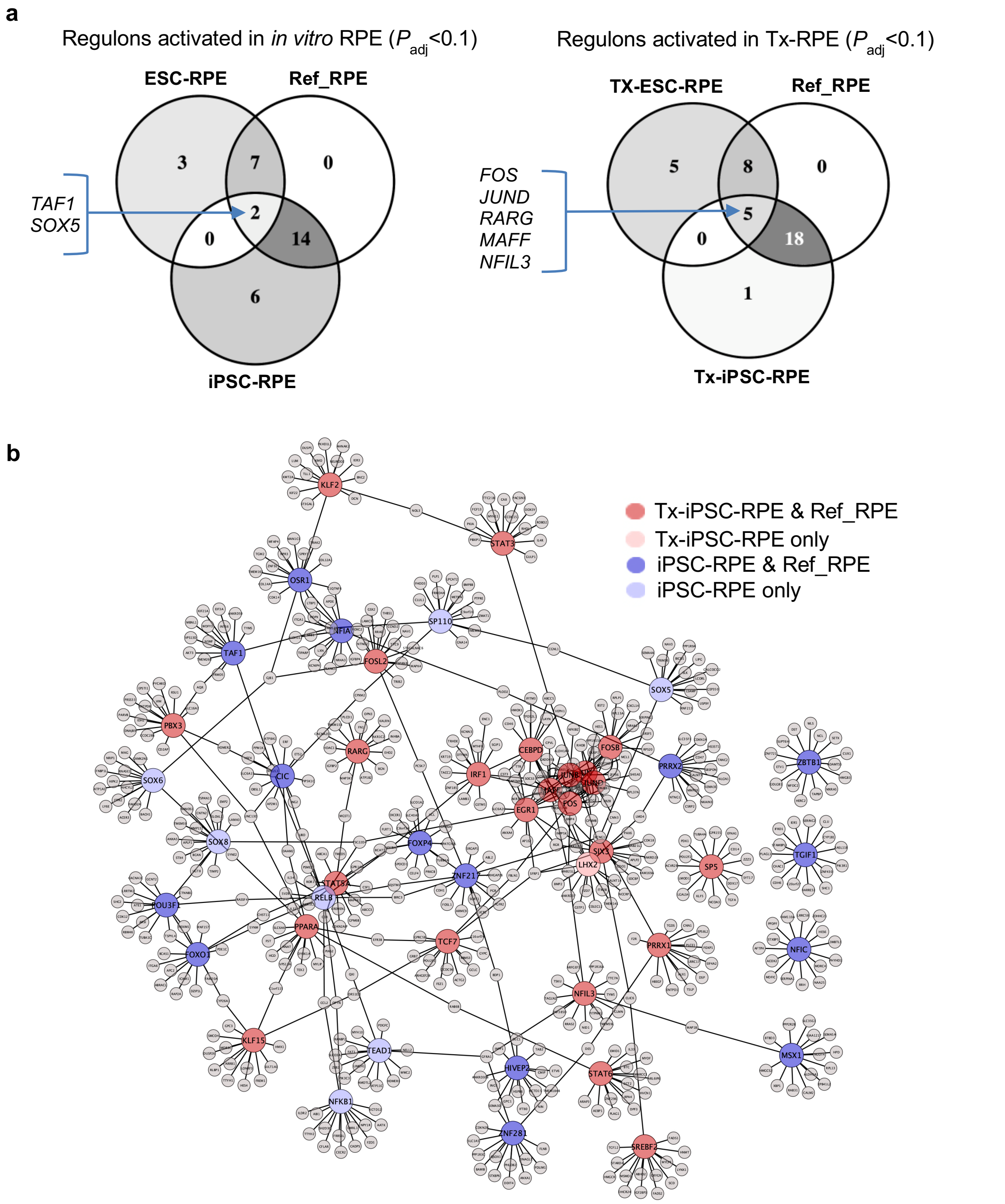

Fig. S10 | Overlapping regulons between RPE lines shows that iPSC-RPE are most similar to Ref_RPE than ESC-RPE, both before and after transplantation. (**a**) Venn diagram showing overlap between regulons concurrently active between both *in vitro* RPE lines (ESC- and iPSC-RPE) and Ref_RPE, or between both post-transplanted RPE lines (Tx-ESC- and Tx-iPSC-RPE) and Ref_RPE. (**b**) Gene regulatory network (GRN) graph showing the regulons concurrently activated between iPSC-RPE, Tx-iPSC-RPE and Ref_RPE.

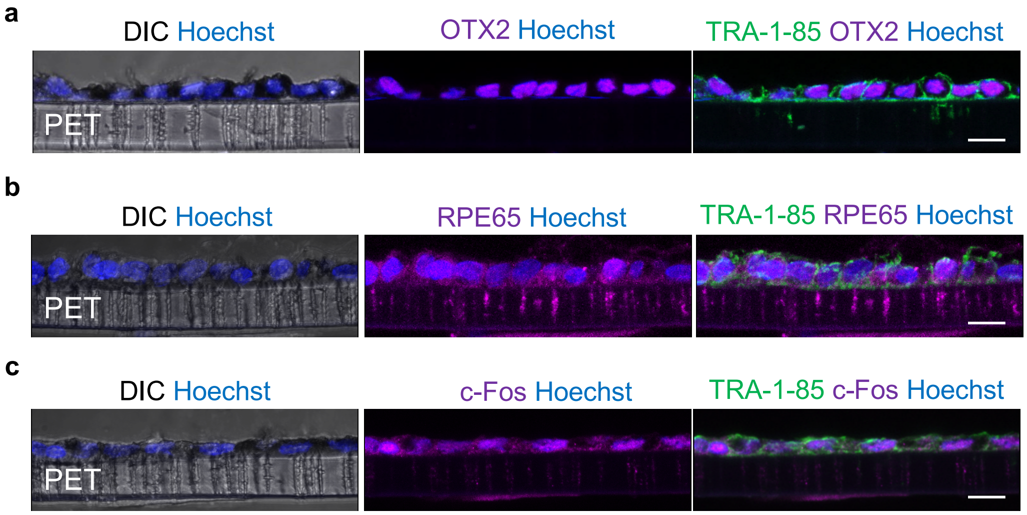

Fig. S11 | *Ex vivo* immunofluorescence of c-Fos expression in transplanted stem cell-derived RPE in rabbit eye. (**a**) Differential interference contrast (DIC) microscopy images showed pigmented (presence of dark granules) cells laid on the PET scaffold, which were positive for human-specific antigen (TRA-1-85, green) and RPE identity transcription factor (OTX2, violet). (**b**) Transplanted RPE also demonstrated positive expression of RPE-specific protein, RPE65 (violet), involved in the visual cycle. (**c**) Nuclear localized c-Fos (violet) expression in transplanted RPE (TRA-1-85, green). Nuclei is labelled in blue using Hoechst stain. Scale bar, 10 μm.

Supporting Information Table

Table S1 | Marker genes used for identification of non-RPE subpopulations.

Retina	Fibroblast	Myoblast	Mesenchymal
NEUROD4	*DPT*	*MYL1*	*DHRS9*
PDE6H	*REN*	*UNC45B*	*NPPB*
STMN4	*C7*	*CHRNA1*	*RAB17*
SYT4	*ASPN*	*APOBEC2*	*TNFRSF11B*
SCG3	*PLN*	*TNNC2*	*NMU*
STMN2		*CKM*	*MYL7*
RTN1		*SMYD1*	
TPH1		*MYOG*	
		MYH8	
		MYBPH	
		MYH6	

Table S2 | Cell counts after applying quality filters

Sample	Pre-filtering	Genes > 500; cells > 3	Rabbit reads < 5%	MT reads < 20%	Remove non-RPE cells
**ESC-RPE**	10526	6723	6723	6600	4882
**Tx-ESC-RPE_1**	5179	4638	2873	2797	221
**Tx-ESC-RPE_2**	14443	11455	2620	2548	50
**Tx-ESC-RPE_3**	12729	12122	11367	11224	1646
**iPSC-RPE**	11695	8027	8027	7758	5890
**Tx-iPSC-RPE_1**	6779	5687	5619	4733	415
**Tx-iPSC-RPE_2**	6553	5360	3416	2853	128
**Tx-iPSC-RPE_3**	5336	3055	18	15	0
**Total**	73240	57067	40663	38528	13232
